# Regulating Copper Nuclearity in Metal–Organic Frameworks for the Hydrogenation of Carbon Dioxide

**DOI:** 10.1002/smtd.70882

**Published:** 2026-08-17

**Authors:** Soufiane Bahou, Chanokporn Kosri, Ashour A. Ahmed, Hesham Mena, Riya Sehrawat, Sebastian Wohlrab, Thanapa Numpilai, Pongtanawat Khemthong, Thongthai Witoon, Kajornsak Faungnawakij, Bunyarat Rungtaweevoranit, Ali Abdel‐Mageed

**Affiliations:** ^1^ Leibniz Institute for Catalysis (LIKAT) Rostock Germany; ^2^ National Nanotechnology Center (NANOTEC) National Science and Technology Development Agency (NSTDA) Pathum Thani Thailand; ^3^ CASUS ‐ Center for Advanced Systems Understanding Helmholtz‐Zentrum Dresden‐Rossendorf e.V. (HZDR) Görlitz Germany; ^4^ Department of Environmental Science, Faculty of Science and Technology Thammasat University Pathumthani Thailand; ^5^ Center of Excellence on Advanced Adsorbents and Catalysts for Carbon Capture and Utilization, Department of Chemical Engineering, Faculty of Engineering Kasetsart University Bangkok Thailand; ^6^ Department of Chemistry, Faculty of Science Cairo University Giza Egypt

**Keywords:** CO_2_ reduction, copper cluster, metal nuclearity, single‐atom catalyst, Zr‐MOFs

## Abstract

Cu‐metalated MOFs, particularly Zr‐based frameworks, are promising CO_2_ hydrogenation catalysts. Tuning Cu nuclearity toward small clusters or isolated atoms is crucial for controlling product selectivity, yet very challenging. One way to achieve this is by adjusting the number of OH/H_2_O pairs on Zr_6_ nodes. While the UiO‐66 framework allows only one anchoring pair per node, the MOF‐808 can host up to six defects via formate substitution, enabling much higher Cu loadings (Cu/Zr_6_ up to 5.2). In addition, we found that the Cu precursor influences Cu nuclearity in MOFs: CuCl_2_ forms mainly isolated sites (Cu_1_), whereas Cu(NO_3_)_2_ promotes clusters (Cu_x_). Cu_x_/MOF catalysts were examined for CO_2_ hydrogenation at the gas/solid and at liquid(gas)/solid interfaces. At the gas/solid interface, Cu_x_/MOF catalysts showed higher activity and selectivity toward methanol, with Cu_x_/UiO‐66 outperforming the Cu_x_/MOF‐808 catalyst, while Cu_1_/MOFs were barely active toward CH_4_ and CO formation. In the liquid phase, both Cu_x_/MOF catalysts were active only for methanol formation. These results, together with detailed structural characterizations using X‐ray absorption and diffuse reflectance FTIR spectroscopy during pretreatment and CO_2_ reduction, electron microscopy, and a number of basic characterizations (N_2_ adsorption, PXRD, DR‐UV‐vis, thermal analyses, and NMR spectroscopy), are discussed in relation to the structure‐reactivity relationships, and are supported by DFT modeling of the Cu_x_ ensembles in both frameworks.

## Graphical abstract

CO_2_ hydrogenation on Cu‐metalated MOFs at gas–solid and liquid–solid interfaces depend strongly on the nuclearity of the Cu, which can be adjusted via the density of defects and the synthesis process. In both reaction regimes, methanol formation is favored on Cu clusters rather than on isolated Cu sites, highlighting the critical role of metal nuclearity in reactivity control.

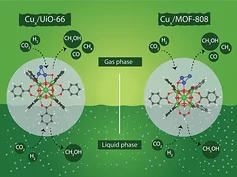

## 1 Introduction

The electronic properties of metal catalysts are strongly altered as metal dispersion increases from the nanometric size range [1, 2] down to the atomic dispersion level [3]. The sub‐nanometric (sub‐nm) regime is particularly unique, representing an intermediate state between nanometric particles and atomically dispersed sites stabilized by oxo‐coordination ligands/surface sites. Herein, clusters composed of a few atoms exhibit molecule‐like characteristics, including pronounced changes in electron density, band gap, and electrical conductivity [4], which together strongly influence adsorption and catalytic behavior. The intrinsic catalytic properties of these tiny metal clusters have been demonstrated for supported Cu clusters in CO_2_ reduction. It was shown that sub‐nm Cu clusters supported on Al_2_O_3_ exhibit enhanced activity for CO_2_ hydrogenation to methanol as cluster size decreases to a few atoms (e.g., Cu_4_) [5, 6]. Similar behavior was reported also for the hydrogenation of dimethyl carbonate to methanol over Cu/CeO_2_ catalysts containing sub‐nm Cu clusters [7]. In contrast, isolated Cu single sites supported on MOFs (e.g., UiO‐66 [8, 9]) or metal oxides (e.g., Cu/CeO_2_ [10]) are primarily active for CO_2_ reduction to CO and show negligible activity toward hydrogenation steps leading to methanol or higher alcohols. This behavior is likely linked to the limited ability of isolated sites to activate hydrogen compared to metal clusters. For CO_2_ hydrogenation to methanol, Cu remains a standout metal due to its optimal binding strength with key intermediates during CO_2_ hydrogenation. However, achieving stable, highly dispersed Cu clusters under reaction conditions remains challenging. Metal cluster sintering is particularly a main cause of deactivation of Cu/ZnO/Al_2_O_3_ catalysts during methanol synthesis [11].

Among several systems, the Cu catalysts based on Cu–ZrO_2_ interface showed distinct activity toward CO_2_ hydrogenation [12, 13]. The interface between ZrO_2_ and metal is an ideal site for CO_2_ adsorption. This is related to the formation of perimeter oxygen defects [14]^,^ which was also demonstrated for Au/ZnO [15, 16, 17] and Ru/ZrO_2_ [18, 19] during CO_2_ hydrogenation to methanol and methane, respectively. In this context, metal clusters are required for hydrogen dissociation and subsequent catalytic transformations. Maximizing metal–metal oxide perimeter sites can yield catalysts with a high density of active sites.

In this contribution, we build on our previous efforts to develop a rational strategy for designing atomically/sub‐nanometric dispersed Cu ensembles, in which the active sites consist of only a few atoms. In this configuration, each atom remains theoretically accessible to reactants. The central challenge lies in achieving such precise control over few‐atom catalyst synthesis. We propose that the structural diversity and well‐defined nodes of metal–organic frameworks can address this challenge. Specifically, controlled Cu nuclearity can be achieved by regulating the number of Cu atoms loaded per metal‐oxide node, an approach supported by prior studies showing that single‐atom Cu catalysts form when one Cu atom or low‐nuclearity clusters (i.e., few atoms) are anchored per metal‐oxide cluster [16, 20, 21]. To control the number of Cu atoms on the cluster, we selected MOFs with different amounts of anchoring sites, that is, OH/H_2_O pairs on the metal oxide cluster. From structural analysis, UiO‐66 and MOF‐808 contain intrinsically one pair of OH/H_2_O per metal oxide cluster. A key distinction between these two frameworks appears, however, in their defect engineering capacity. UiO‐66 allows only one OH/H_2_O pair per Zr_6_ node. MOF‐808 accepts up to six such sites after formate anions replace terminal ligands. This structural arrangement would presumably favor the formation of Cu clusters over isolated single sites. This structural difference has a direct effect on catalytic performance and determines methanol productivity and selectivity. Therefore, we selected these two MOFs for to install Cu atoms on these OH/H_2_O sites. Additionally, the number of Cu atoms can be further tuned by conditions in which Cu ions are loaded on the MOF, particularly the type of Cu precursor.

Understanding and rational optimization of the interaction of these metal ensembles within the structurally flexible MOFs would allow this way to engineer different active motifs suitable for a number of catalytic processes [22], and related applications [23]. Such structural modifications are specifically essential for tuning the selectivity of the CO_2_ reduction reaction, whereas the local geometric and electronic environment of the active sites plays a decisive role in directing reaction pathways, which are sensitive to metal nanostructure (e.g., single sites vs. clusters or nanoparticles) [24]. In this context, Zr‐based MOFs are particularly attractive platforms [25], as they have well‐defined molecular structure and, more importantly, high thermal, chemical, and mechanical stability, which is critical for withstanding the harsh conditions typically required for CO_2_ reduction, including elevated pressures and temperatures [26]. The synthetic strategy and catalytic performance are examined for reactions occurring at gas/solid and liquid(gas)/solid interfaces. These studies are complemented by comprehensive structural characterizations, including operando Cu K‐edge X‐ray absorption spectroscopy and diffuse reflectance FTIR spectroscopy conducted during catalyst pretreatment and CO_2_ reduction. Additional insights are provided by high‐resolution electron microscopy, along with a suite of fundamental characterization techniques such as N_2_ adsorption, powder X‐ray diffraction (PXRD), diffuse reflectance UV–vis spectroscopy, thermal analyses, and NMR spectroscopy. Together, the results of these methods are discussed in the context of elucidating detailed structure–reaction relationships.

## 2 Results and Discussion

### 2.1 Catalyst Synthesis and Basic Characterization

**FIGURE 1:**
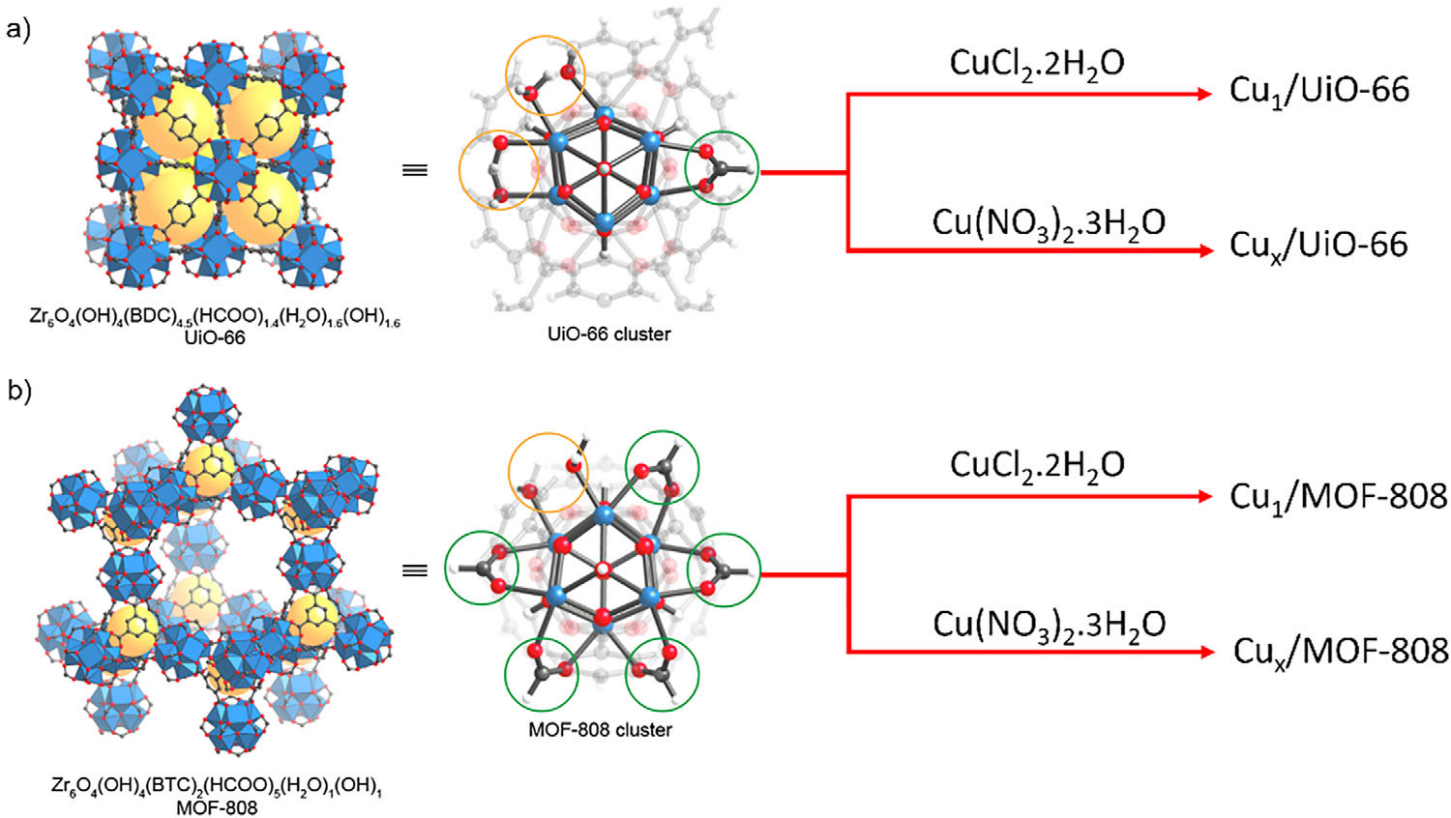
Design and synthesis approach of atomically dispersed Cu catalysts using UiO‐66 (a) and MOF‐808 (b) frameworks to control Cu atomicity. Atom labeling scheme: C, black; O, red; N, green; Cu, orange; Zr, blue polyhedra. Orange spheres represent the space in the tetrahedral cages.

The UiO‐66 and MOF‐808 frameworks were synthesized using a solvothermal method following the reported procedures [9, 16, 20, 27, 28]. To prepare single‐atom catalysts, UiO‐66 and MOF‐808 frameworks were reacted with CuCl_2_·2H_2_O in DMF at 85°C overnight to yield Cu_1_/UiO‐66 and Cu_1_/MOF‐808 catalysts, respectively (see Figure 1). The average numbers of Cu atoms per individual ZrO_x_ node in both MOFs are 1.3 and 1.4, respectively (see Table 1).

**TABLE 1:** Summary of the textural properties of the prepared UiO‐66 and MOF‐808 frameworks and their Cu‐metalated analogues.

Catalyst	BET surface area of as‐synthesized catalysts (m^2^/g)	Pore volume (cm^3^.g^−1^)	Formate/Zr_6_	Cu/Zr_6_	Cu (wt%)
UiO‐66	1405	0.51	1.4	—	—
Cu_x_/UiO‐66	1156	0.48	0	1.70	5.43
Cu_1_/UiO‐66	1268	0.51	0.5	1.3	3.52
MOF‐808	1884	0.83	5.0	—	—
Cu_x_/MOF‐808	1177	0.53	1.9	5.2	12.72
Cu_1_/MOF‐808	1497	0.65	3.5	1.4	4.97

*Cu and Zr loadings were determined from ICP‐OES. The ratios between Zr_6_ and the linker were determined from TGA measurements and ICP‐OES. The ratio between linker and formate ligands were determined from NMR experiments.

**FIGURE 2:**
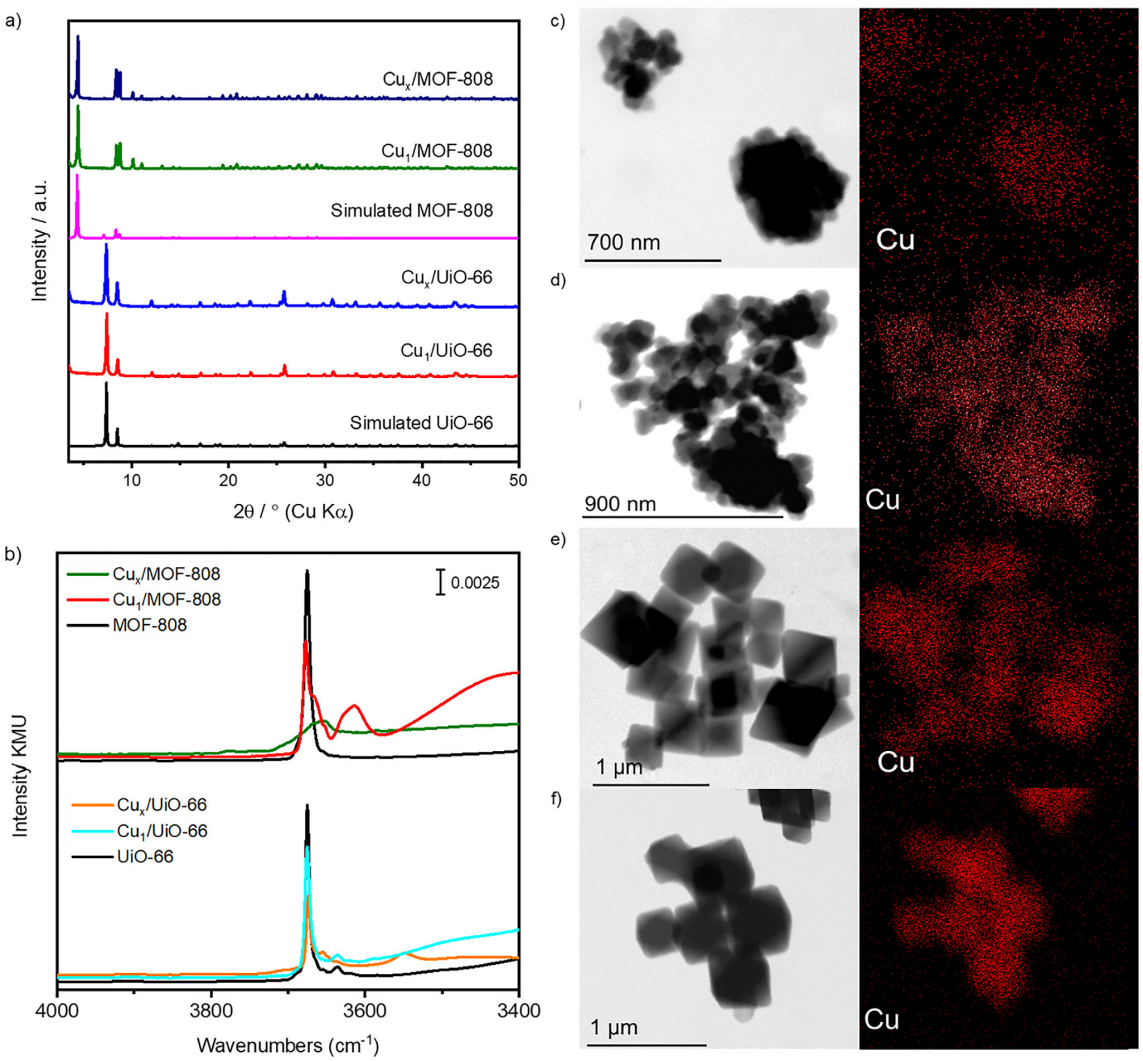
(a) PXRD patterns of the as‐synthesized UiO‐66 and MOF‐808 frameworks and their metalated counterparts, including isolated Cu atoms (Cu_1_/UiO‐66 and Cu_1_/MOF‐808) and Cu clusters (Cu_x_/UiO‐66 and Cu_x_/MOF‐808). (b) FTIR spectra collected on the as‐synthesized MOFs and the metalated counterparts. (c–f) Overview STEM micrographs of as‐synthesized (middle panels) and corresponding Cu EDS elemental maps (right panels) of different Cu‐metalated samples (c: Cu_1_/UiO‐66; d: Cu_x_/UiO‐66; e: Cu_1_/MOF‐808; f: Cu_x_/MOF‐808). EDX elemental maps for different elements (C, O Cu, and Zr) are compared in the Supporting Information for all samples after different reaction phases (see Figures S31–S34).

To increase the number of Cu atoms per ZrO_x_ node to create Cu clusters instead of Cu_1_ sites, the UiO‐66 and MOF‐808 frameworks were stirred in a solution containing Cu(NO_3_)_2_·3H_2_O, triethylamine, and DMF at room temperature overnight, resulting in Cu_x_/UiO‐66 and Cu_x_/MOF‐808 catalysts. The number of Cu atoms per oxide node is 1.7 and 5.2, respectively (see Table 1). The much higher Cu loading per ZrO_x_ oxide node is clearly explained by the larger number of molecular defects introduced into MOF‐808 framework. The structural integrity of the Cu‐metalated MOFs, with respect to the metal‐free MOFs, was studied using PXRD, N_2_ adsorption and ICP‐OES/elemental analyses (see Table 1). First, the XRD of the Cu_x_/UiO‐66 and Cu_1_/UiO‐66 displays a similar pattern compared to the bare UiO‐66, and these show the reflections expected from the simulated UiO‐66 pattern (Figure 2a). In addition, the PXRD patterns of Cu_1_/MOF‐808 and Cu_x_/MOF‐808 catalysts were similar to the bare MOF‐808, which also mimic the simulated MOF‐808 pattern (Figure 2a). The PXRD results of all catalysts indicated that the introduction of Cu, independent of its quantity or the salt used in the synthesis, did not change the structure of the parent MOFs. In addition, there are no diffraction peaks characteristic of CuO_x_ or Cu_x_ species, which implied that the immobilized Cu was incorporated into the framework structure in a highly di spersed state. The diffraction pattern of the thermally treated samples at 200°C under a flow of 10% H_2_/Ar indicated a stable framework structure under a reductive gas atmosphere (see Figure S1).

Afterwards we examined the Cu metalation into the defect sites of the two frameworks using DRIFT spectra. The FTIR spectra under N_2_ flow at 175°C indicate bands around 3715 cm^−^^1^ and a smaller shoulder at 3678 cm^−^^1^ for metal‐free UiO‐66 and MOF‐808. These features are attributed to terminal/defect Zr–OH groups on the Zr‐oxide nodes, which decrease upon Cu metalation of both UiO‐66 and MOF‐808 frameworks (see Figure 2b). This behavior indicates partial consumption and perturbation of these defect hydroxyl groups by the incoming Cu species. In addition, the distinct magnitude and shape of spectra between MOF‐808 and UiO‐66 reflect differences in node geometry, defect density, and site accessibility, which is consistent with site‐specific incorporation of Cu into Zr‐node defect sites.

Next, we examined the porosity of both MOFs and their metalated counterparts using N_2_ adsorption measurements. As shown in Figure S2, UiO‐66 shows higher N_2_ uptake compared to its Cu‐loaded UiO‐66, which indicates partial pore blocking upon metalation by Cu. A similar trend is observed for MOF‐808 in Figure S3, where the incorporation of Cu leads to a decrease in N_2_ uptake relative to the pure framework. As expected, MOF‐808 shows a significantly higher surface area and pore volume than UiO‐66 in all cases (see Table 1), which demonstrates its larger pore structure. However, as highlighted in Figure S4 and Table 1, the prepared MOFs /Cu‐MOFs responded differently to pretreatment. In the case of Cu_x_/MOF‐808, the reduction made a significant impact: the material not only regained its adsorption ability but even outperformed the unreduced MOF‐808. This improvement may be attributed to the reduction of Cu^2+^ species loaded into the framework and the removal of counter anions after reduction (see Figure S4), which matches the higher surface area and pore volume values measured (see Table 1).

Based on the ^1^H NMR analyses of the MOFs and their Cu‐metalated counterparts (Figures S5–S10), elemental analysis (C,H,O,N)/ICP‐OES (Table 1), and thermal gravimetric analysis (Figures S11,S12), it was deduced that the MOF contains approximately one missing BDC per Zr_6_ cluster for UiO‐66 and one missing BTC linker per Zr_6_ cluster/node for MOF‐808. These defect sites are capped by acetate and –OH/–OH_2_ groups. To further investigate the presence of Cu clusters, we conducted high‐angle annular dark‐field scanning transmission electron microscopy (HAADF‐STEM) and EDS measurements on the freshly reduced Cu catalysts (see Figure 2c–f). While the morphology of the UiO‐66 and MOF‐808 is discernible from the HAADF‐STEM micrographs, we can see the Cu distributed through the MOF crystallites. We also cannot see any regions of Cu aggregates or differentiate them from Cu clusters or single atoms for the following two reasons: Very low Z‐contrast between Zr and Cu prevents clear distinction between ZrO_x_ and Cu_x_ clusters (as discussed in [29, 30]). More importantly, multiple Cu nanostructures are assumed to coexist, such as isolated single sites, small clusters (dimers–tetramers), and Cu_x_ particles, making them hard to count and visually distinguish [20, 21, 29]. Additionally, the STEM images show that the morphology of Cu_x_/MOFs is similar to that of Cu_1_/MOFs. This observation aligns with the PXRD results, confirming that the structure remained unchanged after Cu immobilization, while excluding the presence of Cu clusters. The morphology of the MOF microcrystal is also not changed upon exposure to the reduction step after synthesis, as indicated in the TEM data collected after different stages of reaction (Figures S13–S15).

### 2.2 In Situ Cu K‐Edge X‐ray Absorption Spectroscopy

#### 2.2.1 Cu K‐Edge XANES

**FIGURE 3:**
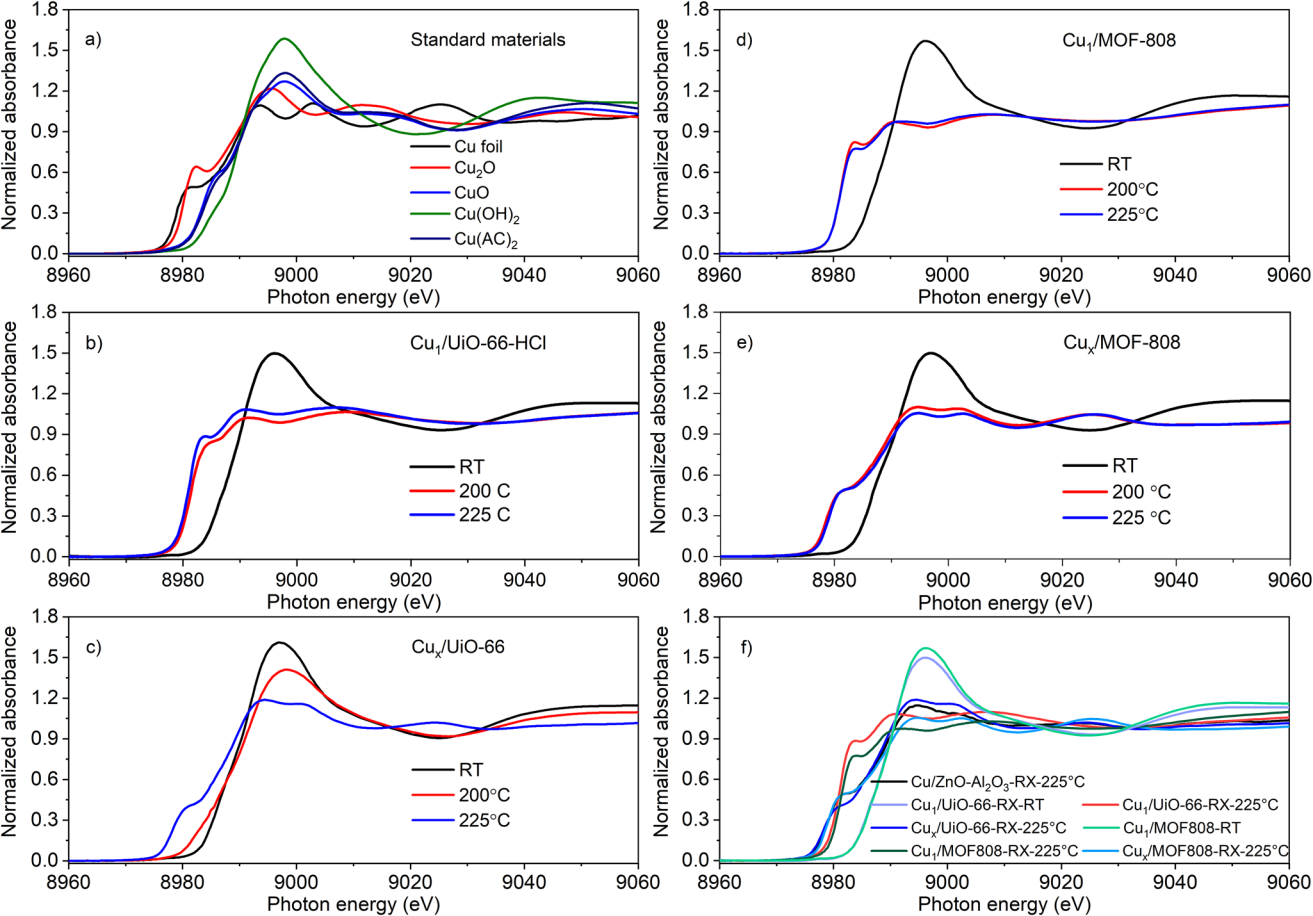
XANES spectra at Cu K‐edge of: (a) different Cu references (Cu metallic foil; Cu_2_O, CuO, Cu(OH), Cu‐acetate (Cu(AC)_2_)); (b) Cu_1_/UiO‐66 collected at room temperature and during CO_2_ reduction at 200°C/225°C; (c) Cu_x_/UiO‐66 at room temperature and during CO_2_ reduction at 200°C/225°C; (d) Cu_1_/MOF‐808 at room temperature and during CO_2_ reduction at 200°C/225°C; (e) Cu_x_/MOF‐808 at room temperature and during CO_2_ reduction at 200°C/225°C; (f) comparison of the XANES selected spectra of Cu_1_ and Cu_x_ MOF‐based catalyst with benchmark Cu/ZnO‐Al_2_O_3_ catalyst recorded during CO_2_ reduction (RX) at 225°C (RT: refers to spectra collected at room temperature of the fresh catalyst).

Next, we studied the evolution of electronic properties and local coordination environment of Cu species encaged into the MOFs before and during the CO_2_ hydrogenation reaction. As a reference for discussion of the collected spectra of all catalysts, we compared in Figure 3a spectra of different Cu references, including metallic Cu (Cu foil), Cu^1+^ (Cu_2_O), and Cu^2+^ references (CuO, Cu(OH)_2,_ and Cu‐acetate (Cu(AC)_2_). First, the comparison of the spectra collected on the Cu_1_/UiO‐66 catalyst during reaction shows clearly a strong reduction of the Cu^2+^‐like state in the fresh sample during reaction. Interestingly, although the electronic state is Cu^1+^‐like, it does not copy the shape of the Cu^1+^ reference (Figure 3a,b). The increase in reaction temperature for these catalysts from 200°C to 225°C also did not affect the electronic state of Cu_1_ sites. For the Cu_1_/MOF‐808 catalyst, we observed similar spectral features that are distinctly different from CuO and Cu_2_O references, although it is more resembling the Cu^1+^‐like state.

We examined the Cu_x_/UiO‐66 catalyst similarly during CO_2_ reduction at 200°C and 225°C. The spectra show that the extent of reduction of Cu_x_ clusters is relatively different from that of Cu_1_ sites in Cu_1_/UiO‐66 catalyst (Figure 3c). Although the shape of the spectra reflects tiny differences from that of the Cu_1_ sites but is still distinctly different from that of the Cu oxide references. The spectra of the Cu_x_/MOF‐808 catalyst showed distinct differences from those of the Cu_1_/MOF‐808 catalyst, but there were no changes upon increasing the reaction temperature (see Figure 3e). For a direct comparison, we show in Figure 3f all spectra of catalysts collected during CO_2_ hydrogenation at 225°C in comparison with the spectra collected on the Cu/ZnO‐Al_2_O_3_ benchmark catalyst under identical conditions. Evidently, the Cu species in the case of the Cu/ZnO‐Al_2_O_3_ catalyst closely resembles the spectral features of the XANES spectra of the metallic Cu foil, indicating a more metallic state, which is not the case of monoatomic Cu sites or Cu_x_ clusters encaged in both MOFs. This represents an intermediate oxidation state closer to the Cu^1+^ species rather than the Cu^2+^ state.

#### 2.2.2 Cu K‐Edge EXAFS

**FIGURE 4:**
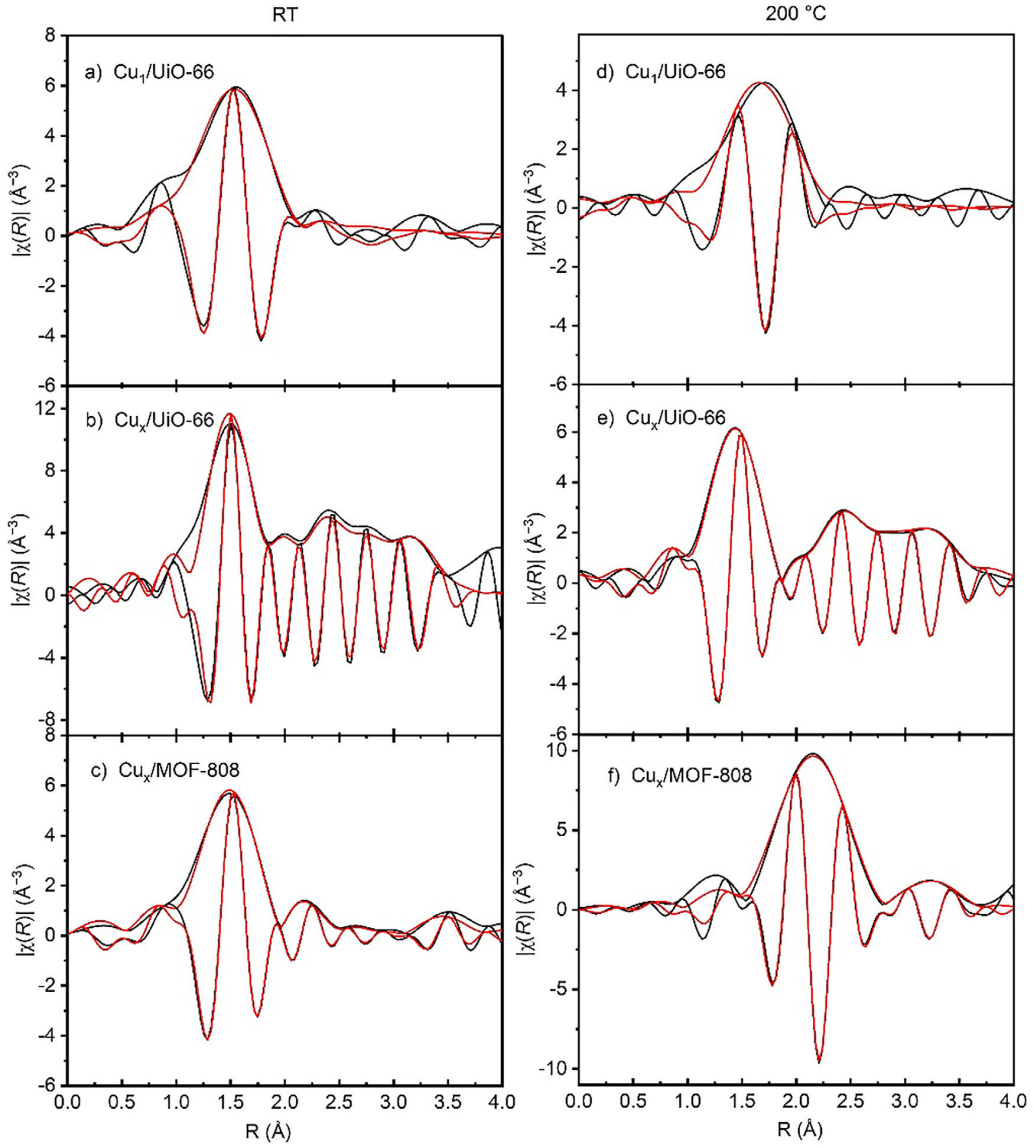
Cu K‐edge FT‐EXAFS spectra for the Cu_1_/UiO‐66 (a, d), Cu_x_/UiO‐66 (b, e), and Cu_x_/MOF‐808 catalysts (c, f). Data on the left panels (a–c) are for the pristine catalysts, while data on the right panels (d–f) are collected during CO_2_ hydrogenation.

Next, we examined the local coordination of the Cu_x_ clusters formed in the two frameworks, as well as the Cu^1^ sites in the UiO‐66 framework, by analyzing the EXAFS data during the activation step and subsequent CO_2_ hydrogenation (Figure 4). First, the data collected for the Cu_1_/UiO‐66 catalyst at RT show that the Cu in the first shell is coordinated by O and Cl atoms, appearing at distances of 1.94 ± 0.05 Å and 2.23 ± 0.06 Å. Note that the Cl backscattering shell would refer to residual chloride anions left after the synthesis of Cu_1_/UiO‐66 [31]. The Cu‐O1 distance is measurably longer than the distance in the bulk Cu_2_O (1.85 Å) [32] and also in the longer range of those in CuO (1.9–2.05 Å) [33]. Upon reduction the catalyst showed only coordination to oxygen at distances of 1.99± 0.04 and 2.49 ± 0.06 Å respectively for first (Cu–O_1_) and second (Cu–O_2_) oxygen coordination shell (see Table 2) [34, 35] Note that there is no Cu‐Cu coordination observed after reduction, and the Cu–O CNs are rather small, characteristic for the presence of Cu single sites [20, 30].

**TABLE 2:** Summary of data fit parameters of Cu K‐edge EXAFS data on Cu/MOF catalysts.

Ab‐Sc pair^a^	CN^b^	R^c^	DWF^d^	R‐factor^e^
**1. Cu_1_/UiO‐66 (fresh as prepared at RT)**				
Cu–O	3.2 ± 1.4	1.94 ± 0.05	0.0080 ± 0.0081	0.012
Cu–Cl	1.1 ± 0.5	2.23 ± 0.06	0.0090 ± 0.0109	
**2. Cu_1_/UiO‐66 (in 10% H_2_ at 200°C)**				
Cu–O_1_	2.1 ± 0.1	1.99 ± 0.04	0.0065 ± 0.0040	0.025
Cu–O_2_	1.9 ± 0.1	2.49 ± 0.03	0.0121 ± 0.0086	
**3. Cu_x_/UiO‐66 (fresh as prepared at RT)**				
Cu–O_1_	4.2 ± 1.6	1.95 ± 0.03	0.0015 ± 0.0044	0.023
Cu–O_2_	2.1 ± 0.8	2.23 ± 0.07	0.0029 ± 0.0068	
Cu∙∙∙Cu_1_ (paddle wheel)	1.0 ± 0.4	2.55 ± 0.05	0.0045 ± 0.0065	
Cu∙∙∙Cu_1_ (O_x_)	0.5 ± 0.2	2.84 ± 0.06	0.0004 ± 0.0074	
Cu∙∙∙Cu_2_(O_x_)	1.0 ± 0.4	3.55 ± 0.05	0.0010 ± 0.0059	
**4. Cu_x_/UiO‐66 (in 10% H_2_ at 200°C)**				
Cu–O_1_	2.2 ± 0.3	1.90 ± 0.01	0.0021 ± 0.0014	0.004
Cu–Cu_1_(O_x_)	0.9 ± 0.5	2.80 ± 0.02	0.0056 ± 0.0026	
Cu–Cu_1_(M)	0.4 ± 0.5	2.52 ± 0.03	0.0055 ± 0.0042	
Cu–Cu_2_ (M)	0.9 ± 0.5	3.47 ± 0.02	0.0006 ± 0.0022	
**5. Cu_x_/MOF‐808 (fresh as prepared at RT)**				
Cu–O_1_	6.1 ± 2.9	2.00 ± 0.03	0.0098 ± 0.0058	0.003
Cu–O_2_	3.6 ± 1.7	2.32 ± 0.05	0.0105 ± 0.0041	
Cu–Cu_1_(M)	1.2 ± 0.6	2.63 ± 0.05	0.0084 ± 0.0062	
Cu–Cu_1_(O_x_)	1.2 ± 0.6	2.86 ± 0.07	0.0111 ± 0.0092	
Cu–Cu_2_ (M)	2.4 ± 1.2	3.56 ± 0.07	0.0182 ± 0.0096	
**6.Cu_x_/MOF‐808 (in 10% H_2_ at 200°C)**				
Cu–O_1_	2.0 ± 0.3	1.95 ± 0.05	0.0365 ± 0.0094	0.014
Cu–Cu_1_(M)	8.0 ± 1.2	2.50 ± 0.03	0.0134 ± 0.0016	
Cu–Cu_2_(M)	4.0 ± 0.6	3.50 ± 0.03	0.0160 ± 0.0039	

^a^ Ab = absorber; Sc = scatterer (numbers 1, 2 and 3 refer to first and second coordination shells in oxidic CuO_x_ and metallic Cu_x_ species; M: metallic shell; Ox: oxidic shell /Cu–O–Cu).

^b^ Coordination number.

^c^ Distance (Å).

^d^ Debye‐Waller factor (Å^2^).

^e^ A measure of mean square sum of the misfit at each data point. Fit range: 3 < *k* < 10 Å^−1^; 1 < *R* < 4 Å; Fit window: Hanning.

For the Cu_x_/UiO‐66 catalyst measured at RT (see Figure 4b), looking at the first coordination sphere, we found out that Cu atoms are coordinated by both oxygen (at 1.95± 0.03 Å with CN of 4.2±1.6) and Cu (at 2.55± 0.05 Å with CN of 1.0±1.4). Note that the Cu–Cu first shell CN is rather very small compared to that of Cu–O, which hints at a more oxidic Cu cluster that co‐exists with other Cu sites or otherwise is partially metallic (see Table 2). The as‐prepared Cu_x_/UiO‐66 catalyst exhibits higher O coordination, which suggests the presence of multinuclear or cluster‐like CuO_x_ nanostructured environments. Clear Cu–Cu contributions, indicated by backscattering at multiple shells, reveal the formation of paddle‐wheel motifs [36] or small Cu aggregates [35]. Interestingly, the presence of a significant fraction of binuclear Cu (paddle‐wheel) nanostructure agrees with our earlier findings based on DFT modelling and in situ DR‐UV‐vis spectra for the Cu metalated UiO‐66 framework [9, 37]. Additionally, we note that the Cu–O_1_ bond distance for the Cu_x_/UiO‐66 catalyst is comparatively shorter than that in the Cu_1_/UiO‐66 catalyst, suggesting the presence of structurally distinct Cu species for the differently prepared catalysts.

For the Cu_x_/MOF‐808 catalyst, we observe significant differences compared to the Cu_x_/UiO‐66 catalyst. In the fresh, as‐prepared catalysts, the Cu–O bond distance (referring to Cu–O_1_) is 2.00 ± 0.03 Å, which is notably longer than that observed for Cu_x_/UiO‐66. Moreover, a larger Cu–O coordination number is observed (see Table 2). This suggests either the presence of larger CuO_x_ particles or stronger interactions between Cu and oxygen ligands within the MOF‐808 nodes. The presence of multiple Cu–Cu contributions indicates the presence of multinuclear Cu clusters. Interestingly, this observation agrees well with a remarkable red‐shift of the UV‐vis absorption for this catalyst, which hints at the formation of rather bigger Cu_x_ clusters compared to the Cu_x_/UiO‐66 catalyst (see discussion below). In addition, the data fits reveal strong contributions from both short and long Cu–Cu distances, hinting at the presence of less dispersed metallic Cu_x_ and CuO_x_ species. Overall, Cu is less dispersed in MOF‐808 than in the UiO‐66 framework. After reduction, the Cu–O coordination number decreases to approximately 2, while the Cu–Cu coordination number for the first shell increases significantly to 8. This indicates extensive reduction of CuO_x_ species accompanied by decreased dispersion of Cu_x_ species.

In total, EXAFS analysis shows that Cu coordination strongly depends on the MOF framework and synthesis method: Cu_1_/UiO‐66 contains isolated single sites with no Cu–Cu interactions, Cu_x_/UiO‐66 shows partially aggregated but well‐dispersed oxidic and clustered Cu species, and Cu_x_/MOF‐808 exhibits higher Cu–O coordination with strong Cu–Cu interactions, indicating larger, less stable aggregates.

### 2.3 In Situ DR‐UV‐Vis Spectroscopy

**FIGURE 5:**
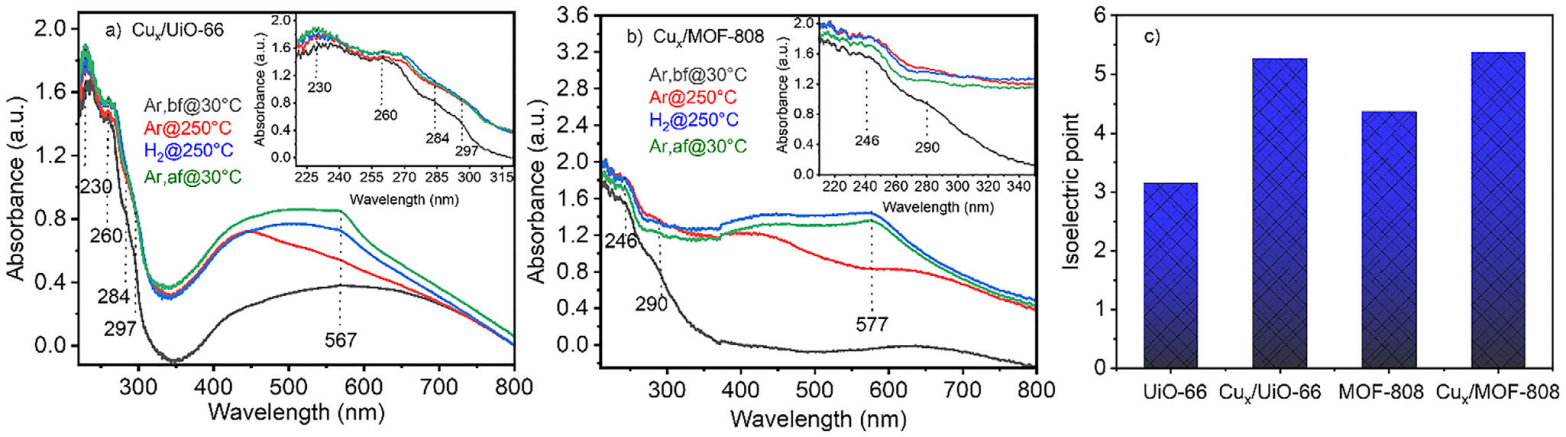
Operando DR‐UV‐Vis spectra recorded for Cu_x_/UiO‐66 (a) and Cu_x_/MOF‐808 (b) catalysts under different conditions: Ar, before heating at 30°C (black line); Ar at 250°C (red); 10% H_2_/Ar at 250°C after 2 h of exposure (green); and Ar, after heating at 30°C (blue) again. (c) Isoelectric point (IEP) of the unmetalated (UiO‐66 and MOF‐808) and the metalated (Cu_x_/UiO‐66 and Cu_x_/MOF‐808) MOFs derived from the study of pH dependence of the isoelectric point (see data in Figure S18).

Next, we used DR‐UV‐vis spectroscopy during the reduction step to gain insight into the electronic and structural properties of the Cu_x_/MOF catalysts (Figure 5a,b). For Cu_x_/UiO‐66, before H_2_ reduction, the absorption peaks appearing at 230, 260, 284, and 297 nm represent mainly the charge transfer transitions associated with O^2–^ ligands and Cu^2+^ species, reflecting oxidized Cu states. A similar observation was reported in previous works [9, 30]. According to UV‐vis characterization of zeolite‐encaged Cu materials, the three maxima at 239, 267, and 297 nm are attributable to Cu^1+^‐like states [38, 39]. Note that there are no signals after 300 nm, which indicates that no metallic Cu clusters are formed and only Cu exists in oxidized states. After the reduction step, an additional absorption band is observed around the 567 nm region, indicating plasmon resonance of small Cu clusters, which agrees with our previous studies supported by DFT simulations of Cu molecular motifs encaged into the UiO‐66 framework (see [9, 37] and discussion therein).

Based on the EXAFS data discussed above (see Table 2), Cu_x_/UiO‐66 has Cu–Cu coordination numbers of approximately 0.9 to 1.0, corresponding to small paddle‐wheel clusters composed of only a few Cu atoms; we conclude that the peak at 567 nm appears due to these sub‐nanometric Cu clusters and provides experimental evidence that this visible absorption arises from metallic cluster formation. Additionally, we did not observe characteristic signals for Cu^2+^ species while the peaks in the 230–297 nm region still existed after reduction. This indicates that some oxidized Cu species remain stable onto the ZrO_x_ clusters of the UiO‐66 framework.

In Cu_x_/MOF‐808, Cu^+^ signals are less prominent, while peaks at 246 and 290 nm are observed, which are likely associated with π → π* transitions of the BTC linker and metal–ligand coordination. At 250°C under Ar flow (red spectrum), an increase in total absorbance suggests structural changes, possibly due to dehydration or desorption of residual solvent from the micropores. After 2 h of reduction at 250°C (blue spectrum), absorbance further increases, particularly in the visible region, with the appearance of a new band around 577 nm, indicating Cu cluster formation. This peak is red‐shifted compared to that observed in Cu_x_/UiO‐66 (Figure 5a,b). This shift may be attributed to the formation of relatively larger Cu clusters [40], or to an increased density of neighboring nanoclusters within the framework, which can sometimes lead to aggregation of nanoparticles or clusters. The first proposal (i.e., red‐shift => larger Cux clusters) agrees quite well with the EXAFS data discussed later, which indicated the formation of larger Cu clusters after reaction (see discussion later). Therefore, in good agreement with EXAFS data, the DR‐UV‐Vis results reveal that Cu species in Cu_x_/MOFs dynamically evolve under thermal and reductive conditions, forming Cu clusters while retaining some oxidized states in both frameworks. This behavior suggests that thermal reduction enhances their catalytic potential for CO_2_ hydrogenation.

Based on the spectroscopic characterization presented for both catalysts, supported by our earlier DFT computational investigations of different Cu nanostructures encaged within the UiO‐66 framework [9, 37], we have simulated the energetically most stable Cu clusters in both MOFs, namely MOF‐808 and UiO‐66. The DFT simulations feature the anchoring of a Cu_3_ cluster on the molecular defects of the Zr‐oxide nodes of both frameworks (see Figure S16). For comparison, we are additionally showing the optimized Cu_1_ sites encaged in both MOFs (see Figure S17). We note that other nanostructures may exist; however, based on previous work, very small clusters, particularly Cu_3_, are more relevant to the catalytic activity of these Zr‐based frameworks.

### 2.4 Zeta Potential Measurements

To gain further insight into the surface charge dynamics of the solid catalyst, we examined the pH‐dependence of zeta potential of the Cu_x_/MOF catalysts [41]. Based on pH‐Zeta potential curves (see Figure S18), we derived the isoelectric points (IEPs) of respective materials (see Figure 5c). UiO‐66 exhibited an IEP of 3.1, indicating a predominantly negative surface charge at neutral pH. Upon copper incorporation (Cu_x_/UiO‐66), the IEP shifted to 5.3, suggesting that copper species located at defect sites contribute to a more positive surface charge. A similar trend was observed for MOF‐808, where the IEP increased from 4.4 to 5.4 following copper addition. These shifts confirm the successful incorporation of positively charged copper species, which alter the surface charge distribution. The increased IEP also reflects the role of copper cations in filling defects or coordination sites, thereby reducing the negative charge typically associated with missing linkers or terminal hydroxyl groups. Such surface modifications not only affect the colloidal stability of the MOFs but may also enhance their interactions with negatively charged species or intermediates during CO_2_ reduction.

### 2.5 CO_2_ Reduction on Cu‐MOFs

CO_2_ reduction was tested in two different reaction regimes. First is the standard test under a flow of CO_2_/H_2_ (1:3) reaction atmosphere, and the second is in liquid phase by pressurizing CO_2_/H_2_(1:3) reaction gas mixture into an autoclave reactor containing the catalyst dispersed in liquid water (gas(liquid)/solid interface).

#### 2.5.1 CO_2_ Hydrogenation from Gas Phase (Gas/Solid Interface)

To benchmark the catalytic performance of Cu‐MOF catalysts, we used a commercial Cu/ZnO‐Al_2_O_3_ catalyst (see PXRD patterns in Supporting Information, Figures S19,S20). This benchmark catalyst was tested under identical conditions used to evaluate the catalytic performance of Cu/MOF catalysts. First, we examined the activity of Cu‐single‐atom based catalysts (Cu_1_/UiO‐66, Cu_1_/MOF‐808) at 20 bar and 200°C. Both catalysts were almost completely inactive for CO_2_ hydrogenation under these reaction conditions. By increasing the pressure up to 50 bar, the Cu_1_/UiO‐66 catalyst showed limited activity (CO_2_ conversion of 0.51%) with methane selectivity of 100% (see Figure S21). By increasing the reaction temperature up to 225°C at 50 bar, the CO_2_ conversion increased to ≈2.0% with CO as the main product (see Figure S22). Despite its high selectivity to methane at 200°C, the limited conversion compared to standard catalysts makes it almost inactive toward practical production of methane [42].

Next, we examined the Cu‐cluster‐based catalysts (Cu_x_/UiO‐66 and Cu_x_/MOF‐808) under identical conditions. Before comparing results among different catalysts, we would like to refer to the time‐on‐stream stability of these catalysts at different temperatures for both catalysts. Both the Cu_x_/UiO‐66 catalyst (Figure S23) and the Cu_x_/MOF‐808 catalyst (Figure S24) showed almost unchanged reaction rates after a short induction period, except for a slow and continuous increase in rates at 200°C and 175°C for the Cu_x_/UiO‐66. In connection with these results, we would like to refer to earlier studies by us and others on the catalytic performance of Cu metalated UiO‐66 catalysts in CO_2_ reduction. In these studies [8, 9], catalytic measurements on Cu_x_/UiO‐66 catalysts showed that after a short initial deactivation, the catalysts reach a stable steady state with no further loss of activity, highlighting their potential for future applications.

**FIGURE 6:**
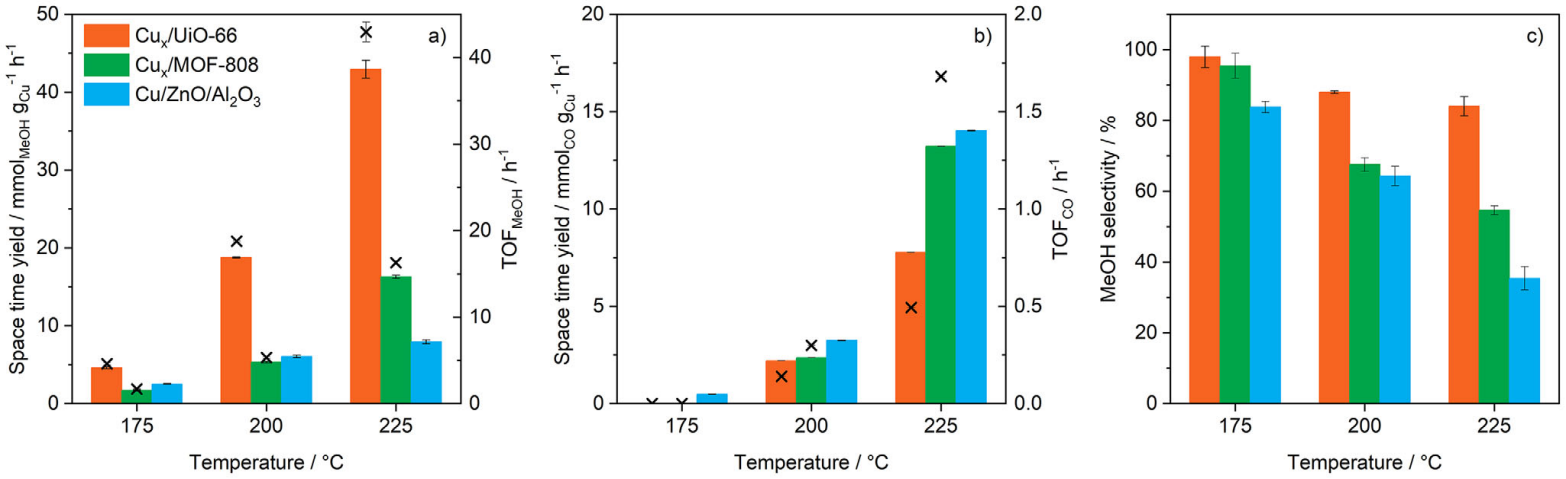
Space‐time‐yield (STY) of (a) methanol formation (represented as mmol_MeOH_ g_Cu_^−1^ h^−1^) and (b) CO formation from CO_2_ at different temperatures and total pressure of 20 bar on Cu_x_/UiO‐66, Cu_x_/MOF‐808, and commercial Cu/ZnO/Al_2_O_3_ catalyst; (c) the selectivity of methanol at different temperatures. Error bars in the figure are calculated as standard deviation from two measurements.

Now, the comparison of all catalysts shall be presented as steady‐state reaction rates. The reaction rate of methanol formation from CO_2_ on Cu_x_/UiO‐66 and Cu_x_/MOF‐808, as well as the benchmark Cu/ZnO/Al_2_O_3_ catalyst, increased with increasing temperature from 175°C to 225°C (see Figure 6a). Additionally, the methanol formation reaction rate for Cu_x_/UiO‐66 is outperforming both the Cu_x_/MOF‐808 and commercial Cu/ZnO‐Al_2_O_3_ catalysts at all reaction temperatures. The behavior of the three screened catalysts was different toward CO formation via RWGS (see Figure 6b). First, only the commercial Cu/ZnO‐Al_2_O_3_ catalyst showed measurable CO formation at 175°C, while the two Cu_x_/MOFs catalysts were completely inactive (see Figure 6b). At 200°C, all catalysts were active for CO formation, but with significantly higher activity for the Cu/ZnO‐Al_2_O_3_ catalyst followed by the Cu_x_/MOF‐808 catalyst and then the Cu_x_/UiO‐66 catalyst showing the lowest CO formation. In total, these results refer to a superior selectivity to methanol for the Cu_x_/Ui‐O66 catalyst at all temperatures, starting from 100% at 175°C going to 85% at 225°C (see Figure 6c).

To further clarify the intrinsic activity of the Cu sites, we calculated the apparent Turnover Frequency (TOF) for methanol formation at 225°C and 20 bar based on the number of moles of Cu per catalyst as basis for normalization. The STY of methanol formation was 1425  g.kg^−1^.h^−1^ for Cu_x_/UiO‐66 and 544 g.kg^−1^.h^−1^ for Cu_x_/MOF‐808. Converting these values to moles of MeOH per kg Cu per hour gives 44.48 mol.kg^−1^.h^−1^ for Cu_x_/UiO‐66 and 16.97 mol.kg^−1^.h^−1^ for Cu_x_/MOF‐808. By dividing the mol MeOH by mol Cu, we obtain TOF values of 2.83 h^−1^ for Cu_x_/UiO‐66 and 1.08 h^−1^ for Cu_x_/MOF‐808. Although the total Cu content in Cu_x_/MOF‐808 (Cu/Zr_6_ = 5.2) exceeds that in Cu_x_/UiO‐66 (Cu/Zr_6_ = 1.7), the TOF rate per Cu atom shows a much greater value for UiO‐66. This indicates that the superior methanol formation activity of UiO‐66 arises from relatively higher intrinsic activity of individual Cu sites trapped into the Ui‐O66 framework rather than from differences in total Cu site density. This trend matches findings from EXAFS data, which show that Cu in UiO‐66 exists mainly in small, highly accessible clusters with a Cu–Cu CN around 0.9–1.0. Larger clusters in MOF‐808 with a Cu–Cu CN up to 8 may contain buried Cu atoms that are less accessible. This is consistent with the activity trends shown in Figure 6, and the corresponding apparent TOF values further highlight the higher intrinsic activity of Cu_x_/UiO‐66. Nevertheless, we note that, due to the difficulty in assigning an exact geometry, and thus the Cu dispersion, the calculated apparent TOF values remain rather qualitative in terms of activity trends.

Finally, we refer to various Cu‑based catalysts reported in the literature that also demonstrate significant methanol selectivity under CO_2_ hydrogenation conditions. Different from our Cu_1_/MOF catalysts, a zeolite‐encaged mononuclear Cu catalyst (Cu@FAU) was found active toward methanol with 89.5% methanol selectivity with space‑time yield of ∼12.8 mmol g_cat_^−1^ h^−1^ at 513 K and 3 MPa [43]. Cu/ZnO‐Al_2_O_3_ catalysts have been found to maintain ∼92%–95% methanol selectivity around 170°C–190°C [44]. The behavior of this catalyst is, however, different from the commercial Cu/ZnO‐Al_2_O_3_ tested in the present study, which showed much lower methanol selectivity (see results in Figure 6). This observation indicates the indispensable need to benchmark catalysts under identical testing conditions for each study. These comparisons indicate that the high methanol selectivity of Cu_x_/UiO‑66 and Cu_x_/MOF‑808 matches state‑of‑the‑art Cu‑based catalysts, especially given the differences in reaction conditions and catalyst architectures.

Overall, CO_2_ hydrogenation tests at the gas/solid interface reveal that Cu single‐atom MOFs are barely active, whereas Cu_x_‐based MOFs, especially Cu_x_/UiO‐66, exhibit superior methanol productivity and selectivity compared to commercial Cu/ZnO‐Al_2_O_3_ catalyst, with increasing temperature enhancing activity while maintaining high methanol selectivity due to suppressed RWGS activity.

#### 2.5.2 CO_2_ Hydrogenation from Liquid Phase (Liquid/Solid Interface)

**FIGURE 7:**
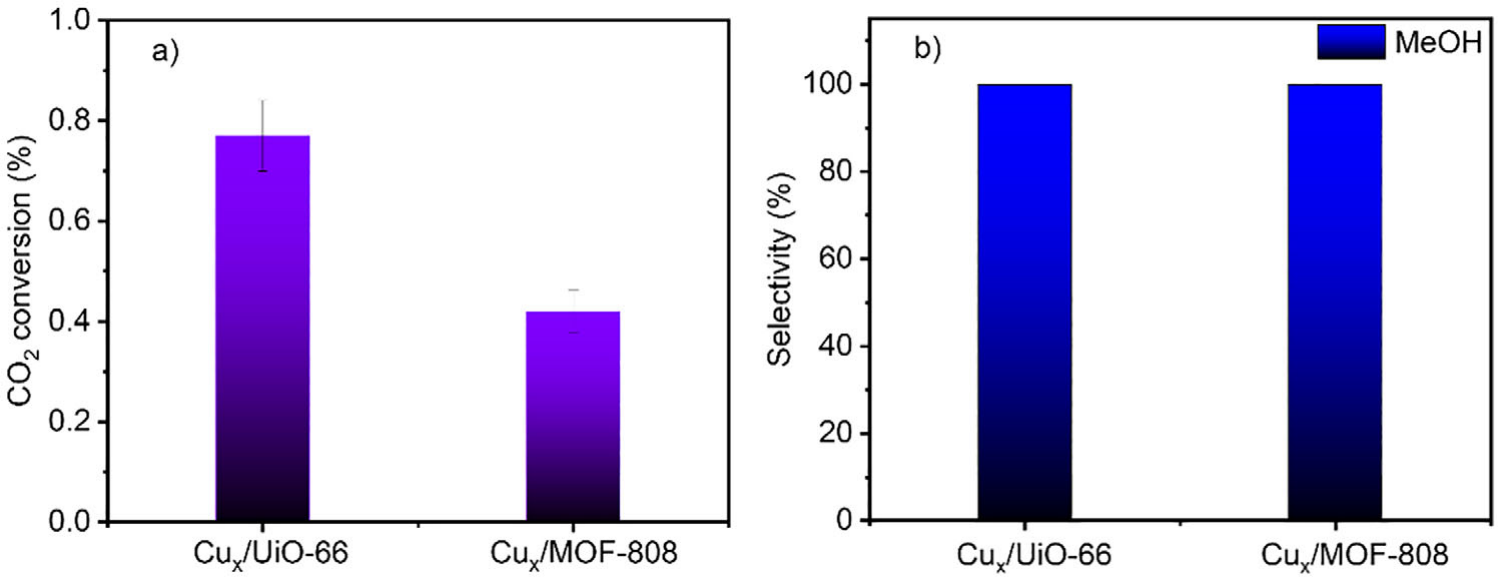
CO_2_ conversion (a) and selectivity (b) on Cu_x_/UiO‐66 and Cu_x_/MOF‐808 catalysts in liquid phase [100 mg of catalyst dispersed in 10 mL of distilled water and pressurized with 5 bar CO_2_ and 15 bar H_2_ at a temperature of 175°C]. See Figures S25,S26 for GC‐MS data. Error bars in the figure are calculated as the standard deviation from two measurements.

As part of our investigation into the correlation between heterogeneous catalyst behavior at gas/solid and gas/liquid/solid interfaces, we explored the CO_2_ hydrogenation performance of Cu_x_/MOF catalysts for CO_2_ reduction in water, which is considered a green solvent. Based on Figure 7a, the CO_2_ conversion of Cu_x_/UiO‐66 reaches around 0.77% and 0.42% for Cu_x_/MOF‐808, which indicates that Cu_x_/UiO‐66 is more active for CO_2_ reduction also in the liquid phase and using water as a green reaction medium, although being a hydrogenation byproduct. Based on analysis of the gas products using the GC, and the liquid phase products using GC‐MS, we quantified product selectivity (see Figure 7b). We detected only methanol for both catalysts under these reaction conditions, hinting at a 100% selectivity to methanol formation. Different from reactions at the gas/solid interface under continuous flow, we do not see CO or CH_4_ formation as side products at 175°C. Note that the relatively low CO_2_ conversion in aqueous medium is primarily attributed to the intrinsically limited solubility of CO_2_ and H_2_ in water, which restricts reactant availability at the catalyst surface, as well as to mass‐transport limitations governing diffusion from the liquid bulk to the solid–liquid interface and/or into the micropores of MOF frameworks. This assumption would be in line with previous studies on CO_2_ conversion on the heterogeneous catalyst CuAu/ZnO at liquid(gas)/solid interface [45, 46]. In addition, it is important to note that suppression of CO formation in the liquid phase may be related to inhibition of the formation of specific intermediates related to CO formation or limiting the desorption of CO formed on the active sites. A deep mechanistic understanding to differentiate these effects and several other scenarios remains, however, open for future systematic studies.

In total, testing identical Cu_x_/MOF catalysts both for the gas‐ and liquid‐phase CO_2_ hydrogenation shows that catalyst performance and selectivity depend on the reaction environment. Gas‐phase conditions favor higher methanol productivity but with lower selectivity, while liquid‐phase reactions in water offer cleaner conversion with full methanol selectivity but with more limited CO_2_ conversion. This emphasizes the potential of liquid‐phase systems for more CO_2_ reduction on solid surfaces.

### 2.6 Characterization of Catalysts Post/During Reaction

To examine the structural stability of the tested catalysts during reaction and get deeper insight into the structure‐reactivity relationship, we applied a combination of methods, either post‐catalytic measurements or during reaction, including PXRD, and STEM‐EDX measurements.

First, we investigated the composition and structural integrity of the catalysts after reaction at different temperatures (175°C, 200°C, and 225°C) using powder X‐ray diffraction (PXRD) and STEM–EDS analyses. Comparison of the PXRD patterns of the gas‐phase spent catalysts with those of the corresponding fresh catalysts (Figures S27–S30) revealed that the crystalline phase of the MOF framework was largely preserved for all samples, except for the spent Cu_x_/MOF‐808 catalyst after reaction at 200°C and 225°C, where noticeable phase changes were observed. For UiO‐66‐based materials (Cu_1_/UiO‐66 and Cu_x_/UiO‐66), the characteristic reflections at 2θ ≈ 7.5° and 8.5° remain visible after reaction but with reduced intensity, which indicates partial loss of crystallinity and preservation of the framework structure intact. However, MOF‐808‐based materials show a much stronger structural degradation. Cu_1_/MOF‐808 shows a clear drop in peak intensity after reaction at 175°C, and the full loss of reflections at 2θ ≈ 4.5°, 8°, and 8.5° after reaction at 200°C and 225°C. This behavior indicates that the higher defect density and Cu loading in MOF‐808 (Cu/Zr_6_ = 5.2) presumably lead to lower structural stability at relatively higher reaction temperatures. Additionally, the formation and growth of larger Cu clusters under reaction conditions, as evidenced by EXAFS, may further contribute to framework destabilization. In contrast, the lower Cu loading and more limited defect density in UiO‐66 help preserve its structural integrity.

STEM images of all spent catalysts after reactions conducted at 225°C showed morphological differences compared to the fresh catalysts, suggesting partial structural or surface reconstruction during catalysis (Figures S31–S34). Despite these morphological changes, EDS mapping confirmed that copper remained homogeneously distributed throughout the spent catalysts, indicating the absence of significant Cu aggregation or segregation under the applied reaction conditions.

### 2.7 Operando DRIFTS During CO_2_ Reduction

**FIGURE 8:**
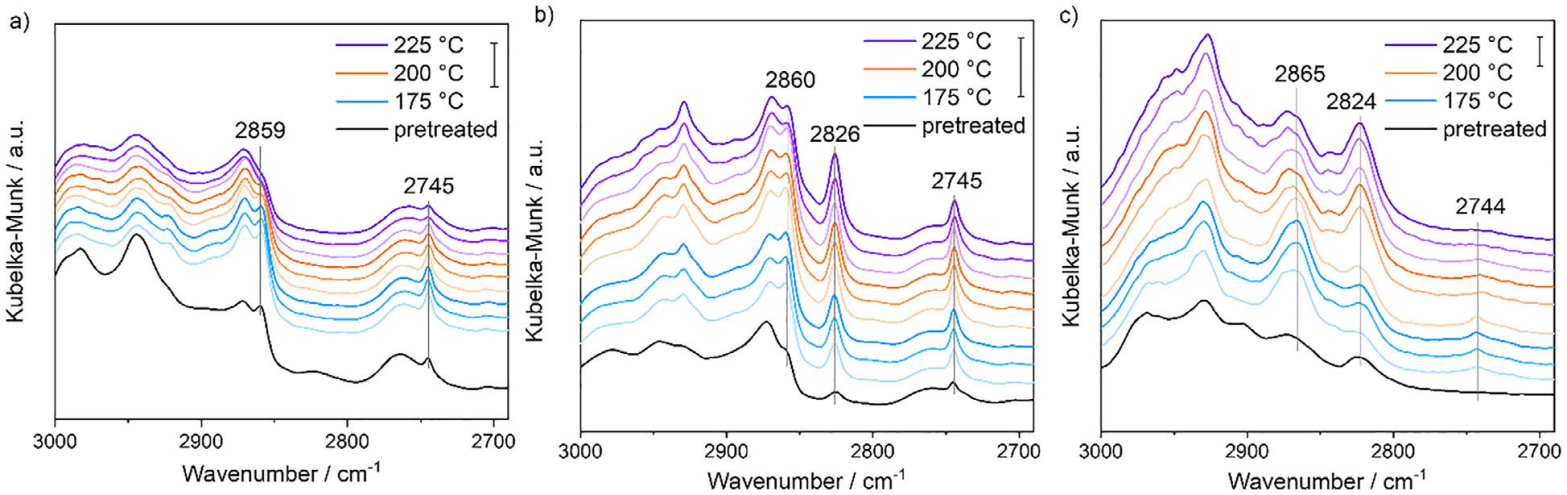
Top layers: Kubelka–Munk converted DRIFT spectra of different catalysts (a) Cu_1_/UiO‐66; (b) Cu_x_/UiO‐66; (c) Cu_x_/MOF‐808) in the spectral range from 3000 and 2600 cm^−1^ after pretreatment (black spectrum) and during CO_2_ reduction (CO_2_/H_2_ = 1:3 at 20 bar) at different temperatures, covering the evolution of OH and formate species (HCOO_ad_).

Finally, operando DRIFTS measurements were used to probe the influence of the framework and type of Cu species (Cu_1_ versus Cu_x_) on dominant surface species forming during CO_2_ hydrogenation on the studied catalysts, which helps to explain differences in reactivity, selectivity, and Cu nanostructure formation in Zr‐based frameworks. We discuss first the spectral region from 3000 to 2600 cm^−1^, showing characteristic signals for expected hydrogenation intermediates of CO_2,_ and compare the 3 catalysts. First, for the Cu_1_/UiO‐66 catalyst, we observed, after pretreatment, features related to residual formate‐like species appearing at 2859 and 2745 cm^−1,^ respectively (see Figure 8a), which are assigned to the C–H stretching and O–C–H bending combination modes of adsorbed formate (HCOO_ad_) [47, 48]. Remaining features are assignable to framework C–H/OH features. Focusing on these two peaks (related to surface formate species), upon exposure to the CO_2_/H_2_ (1:3) mixture at 175°C, these two peaks progressively increased in intensity compared to the background spectrum. The three different spectra at 175°C taken at different times (0 to 30 min on stream) upon exposure to the reaction mixture show a slight decrease with time. Upon increasing the temperature, the intensity of this formate‐like species was found to decrease.

Characteristic features expected for the methoxy group or any other hydrogenation intermediates were not observed during CO_2_ hydrogenation on Cu_1_/UiO‐66; only a small feature at ≈ 2830 cm^−1^ was observed on the pretreated catalyst and disappeared during the reaction. Note that the two features in the spectra in the range from 2910 to 2965 cm^−1^ are assigned to C–H stretch vibration of the organic linkers [49]. For the Cu‐cluster‐based Cu_x_/UiO‐66 catalyst, pretreatment results in a weak baseline similar to that of the Cu_1_/UiO‐66 catalyst. Starting CO_2_ hydrogenation at 175°C and 20 bar, more intense formate‐related peaks at 2860 and 2745 cm^−1^ were observed. At higher temperatures, the intensity of these features remained about unchanged. In addition, we observed a prominent peak at 2826 cm^−1,^ which is assignable to a surface methoxy intermediate, expected to form during the conversion of CO_2_ to methanol. In the case of Cu_x_/MOF‐808, which is also based on a Cu cluster, the evolution of surface formate and methoxy species is even more pronounced. After pretreatment, only weak background features are observed, which develop at ∼2865 and ∼2824 cm^−1^. Compared to UiO‐66‐based catalysts, the higher overall intensity suggests stronger adsorption and/or higher surface concentration of HCOO_ad_ species, likely facilitated by the more open structure and different Zr–O connectivity of MOF‐808, which can stabilize both Cu species and reaction intermediates. Nevertheless, we see a significant decrease in the intensity of all these species upon increasing temperature from 175°C to 225°C during the reaction compared to the more stable behavior on the Cu_x_/UiO‐66 catalyst.

We discuss next the reaction‐relevant carbonyl C–O stretching region, which provides insight into the formation and stabilization of CO‐related intermediates during CO_2_ hydrogenation (Figure S35) and also gives indirect insights into the Cu nanostructures. For the Cu_1_/UiO‐66 catalyst (Figure S35a), the spectrum recorded after pretreatment remains largely featureless in this region except for a shallow peak at 2078 cm^−1^, which is close to peaks attributable to CO adsorption on Cu^1+^ ‐like species [20, 50]. Under CO_2_, a weak but distinct band emerges at 2167 cm^−^^1^ [51], which, at first glance, may be attributed to CO adsorbed on cationic Cu^2+^ species. Considering that CO–Cu^2+^ IR peaks can be observed only at low temperature, down to 100 K, we exclude this assignment. Instead, it may be attributed to co‐adsorption of oxygen species (e.g., from CO_2_ dissociation or an OH group) and CO on Cu^1+^ sites, or otherwise due to traces of gas‐phase CO in the DRIFTS cell. In addition, the weak peak observed at 2078 cm^−1^ before reaction shifted slightly to ∼2076 cm^−^^1^ during reaction, related to CO bound to Cu^1+^ sites [50, 51, 52], which would form from CO_2_ dissociative chemisorption or via RWGS. The weak intensity would agree with the very low CO_2_ conversion observed on this catalyst during catalytic experiments.

For the Cu_x_/UiO‐66 catalyst (Figure S35b), the carbonyl region evolves more clearly upon introducing the reaction mixture. First, we observe a distinct feature at 2159 cm^−^^1^ on the pretreated sample, which decreased with time at 175°C and vanished almost completely at 200°C. This band appears at a lower wavenumber than the band observed on Cu_1_/UiO‐66, but obviously can be explained by the same assignment given above. The peak at 2076 cm^−1^ related to CO adsorption on Cu^1+^ species was also observed. The coexistence of CO species at higher and lower wavenumbers indicates the presence of multiple Cu environments, consistent with EXAFS results showing multinuclear Cu species. The intensity of both types of carbonyl bands remains relatively stable with increasing temperature, pointing to a sustained population of surface CO intermediates under reaction conditions.

In case of Cu_x_/MOF‐808 catalyst (Figure S35c), the pretreated samples showed also the high wavenumber peak but red‐shifted to 2145 cm^−1^. Under reaction conditions at 175°C, this CO_ad_ characteristic feature was replaced by strong gas‐phase adsorption centered around 2145 cm^−^^1^. Notably, upon increasing the reaction temperature from 175°C to 225°C, the intensity of both surface‐bound and gas‐phase CO features decreases, reflecting accelerated consumption of CO intermediates and/or their further hydrogenation or desorption at elevated temperatures. These observations are in good agreement with the higher rate of CO formation in catalytic measurements on the Cu_x_/MOF‐808 catalyst.

Overall, operando DRIFTS clearly demonstrated that the Cu nuclearity and framework environment dictate the dominant surface intermediates (HCOO_ad,_ methoxy, or CO_ad_ species) and, consequently, the reaction pathways observed catalytically. For Cu_1_/UiO‐66, the spectra are dominated by very low surface coverage of formate and CO_ad_ species with no detectable methoxy intermediates, correlated to only very low activity, low CO and CH_4_ formation, and the absence of methanol production. In contrast, both Cu_x_/UiO‐66 and Cu_x_/MOF‐808 exhibit pronounced formate and methoxy signatures under reaction conditions, directly correlating with their ability to produce methanol and CO. Notably, the stronger and more persistent methoxy features on Cu_x_/UiO‐66 are consistent with its markedly higher methanol selectivity and space–time yield compared to Cu_x_/MOF‐808 underscoring the critical role of the UiO‐66 framework in steering Cu clusters toward efficient methanol synthesis pathways.

## 3 Summary and Conclusion

In this work, we developed a rational approach to investigate the synthetic control of Cu nuclearity (isolated single sites and Cu_x_ clusters) into Zr‐based frameworks (UiO‐66 and MOF‐808) and its impact on CO_2_ hydrogenation at gas/solid and Liquid/solid interfaces. The main findings can be summarized in the following points:

- Based on PXRD, electron microscopy, and N_2_ adsorption results, all Cu‐modified UiO‐66 and MOF‐808 samples maintained the parent MOF structures without detectable CuO_x_ peaks, which demonstrates successful Cu incorporation and structural stability upon thermal treatment at 200°C under H_2_/Ar. In addition, this reflects the presence of Cu in a highly dispersed state far below the detection limit of PXRD (<2 nm). Cu loading was enhanced in MOF‐808 through the engineered increase of the number of molecular defects per Zr_6_ oxide node compared to the UiO‐66 framework.
- Catalytic CO_2_ hydrogenation studies were carried out on  the prepared materials both in gas and liquid phases. In the gas phase, catalysts showed higher methanol productivity with some by‐products, while liquid‐phase offer cleaner conversion with full methanol selectivity, which emphasizes the promise of aqueous‐phase systems for sustainable CO_2_ reduction. In both cases, and benchmarked to a commercial Cu/ZnO‐Al_2_O_3_ catalyst, the Cu_x_/UiO‐66 catalyst showed higher activity and selectivity toward methanol formation.
- Post‐reaction PXRD results confirmed the overall structural integrity of the Cu/MOFs catalysts, with only Cu_x_/MOF‐808 showing signs of framework changes at elevated temperatures, which was attributed to the relatively high Cu loading in this catalyst. Additionally, STEM‐EDX results after reaction revealed preserved homogeneous Cu dispersion despite changes in the materials.
- DR‐UV‐vis spectroscopy and Cu K‐edge XANES revealed that Cu_x_/MOF catalysts show subtle changes in oxidation states and structural rearrangements of Cu species under reducing conditions (either during treatment or reaction), whereas small Cu clusters form, in the presence of co‐exisiting oxidized Cu species.
- EXAFS analysis revealed the formation of sub‐nanometric Cu_x_ clusters on both frameworks (Cu_x_/UiO‐66 and Cu_x_/MOF‐808). Under reductive conditions, Cu_x_ species in UiO‐66 are significantly more stable than in MOF‐808, where an increase in the Cu–Cu coordination shell suggests cluster growth during the reaction. In contrast, Cu_1_/UiO‐66 consists of isolated Cu atoms bonded to oxygen, with no cluster formation, and remains stable under CO_2_ hydrogenation conditions.
- Operando DRIFTS experiments showed that the Cu_1_/UiO‐66 catalyst  displayed only low coverage of formate species and no other intermediate compared to Cu_x_/UiO‐66 and Cu_x_/MOF‐808 catalysts which showed strong IR signals for formate, methoxy and CO_ad_ intermediates. This is attributed to the presence of Cu_x_ clusters enhancing H_2_ activation and the sequential hydrogenation of CO_2_ intermediates, consistent with the catalytic tests.

In conclusion, by utilizing structurally different but well‐defined Zr‐based frameworks (UiO‐66 and MOF‐808), we demonstrated that the number of Cu atoms per ZrO_x_ oxide node can be controlled through the change of the number of defects, which together with the type of Cu precursor, can help to control Cu nuclearity (Cu_1_ vs. Cu_x_ species). This helps ultimately in steering the catalytic behavior of Cu/MOF catalysts in CO_2_ hydrogenation. Notably, MOF‐supported Cu single sites, independent of MOF structure (porosity), exhibit negligible activity toward CO_2_ hydrogenation, except for a very limited tendency for CO and/or CH_4_ formation. In contrast, MOF‐supported Cu_x_ clusters show significant activity toward methanol formation, with UiO‐66–based catalysts being more selective toward methanol than MOF‐808–based catalysts. Interestingly, both catalysts showed only methanol formation in the liquid phase using water as a green solvent with complete suppression of any gas byproducts (CO or CH_4_). These differences in structure and activity reflected differences in the adlayer during reaction, where methanol formation correlated well to surface formate and methoxy intermediates under reaction. These findings underscore the importance of controlling Cu nuclearity and MOF structure to achieve improved activity and selectivity in CO_2_ reduction.

## 4 Experimental Methods

### 4.1 Materials Synthesis

#### 4.1.1 Synthesis of MOFs

For UiO‐66 synthesis, 2.5 g of ZrCl_4_ (99.5%; ACROS) and 2.46 g of H_2_BDC (99.0%; TCL Chemicals) were dissolved in 300 ml of dimethylformamide (DMF ‐ 99.8% AR grade; RCL Labscan) in a 500 mL bottom flask. Afterwards 20 ml of 12 M HCl (AR grade; Labsca) was added to the solution dropwise. The reaction flask was sealed and placed in a 85°C isothermal oven overnight. UiO‐66‐HCl was collected using centrifugation (10,000 rpm, 5 min), immersed/filtered in DMF (45 mL × 3) over a 24 h period and in acetone (45 mL × 3) for another 24 h period. Finally, UiO‐66‐HCl powder was filtered out and was then dried under dynamic vacuum overnight at room temperature.

For MOF‐808, the synthesis procedure was similar to that of UiO‐66‐HCl. In a 500 mL media bottom flask, 5.82 g of ZrOCl_2_·8H_2_O (98%; ACROS) and 1.26 g of 1,3,5‐benzenetricarboxylic acid (TCL Chemicals) were dissolved in DMF (180 mL), and 180 ml of formic acid (98‐100%; Merck) was added sequentially to the solution. The reaction flask was sealed and placed in a 100°C isothermal oven for 3 days. MOF‐808 was collected using a centrifuge (10,000 rpm, 5 min), immersed in DMF (45 mL × 3) over a 24 h period, in DI water (45 mL × 3) over a 24 h period, and in acetone (45 mL × 3) over another 24 h period. Finally, the MOF‐808 was dried under dynamic vacuum overnight at room temperature.

#### 4.1.2 Loading Cu Into MOFs

For the preparation of Cu_x_/UiO‐66 and Cu_x_/MOF‐808 catalysts, 3.3 g of Cu(NO_3_)_2_·3H_2_O (99.0‐100; Sigma‐Aldrich) was dissolved in DMF (225 mL) in a 500 mL media bottle. Afterwards, 1.25 ml of Triethylamine (TEA 99.5%; Loba Chemie) was added dropwise while the solution was stirring. The desired support (1 g) was added sequentially, and the suspension was sonicated until the UiO‐66‐HCl powder was dispersed. Then the suspension was stirred overnight at room temperature with a stirring speed of 600 rpm. Cu_x_/UiO‐66 or Cu_x_/MOF‐808 was collected using a centrifuge (10,000 rpm, 5 min) and immersed in DMF (45 mL × 3) over a 24 h period, in DI water (45 mL × 3) over a 24 h period, and in acetone (45 mL × 3) over another 24 h period. Finally, Cu_x_/UiO‐66 or Cu_x_/MOF‐808 was dried under dynamic vacuum overnight at room temperature.

For the preparation of Cu_1_/UiO‐66 and Cu_1_/MOF‐808 catalysts, 540 mg of CuCl_2_·2H_2_O (99.0% Sigma‐Aldrich) was dissolved in DMF (9 mL) in a 20 mL vial screwed with PTFE tape. The desired support (600 mg) was added to the solution and sonicated until all MOFs dispersed. The suspension was heated in 85°C isothermal oven overnight. Cu_1_/UiO‐66 or Cu_1_/MOF‐808 was collected using a centrifuge (10,000 rpm, 5 min). It was immersed in DMF (45 mL × 3) over a 24 h period and in acetone (45 mL × 3) over another 24 h period before being dried under dynamic vacuum overnight at room temperature.

### 4.2 Materials Characterization

#### 4.2.1 Powder X‐ray Diffraction (PXRD)

Powder X‐ray diffraction patterns (PXRD) were recorded on the powder samples using a Bruker D8 Advance diffractometer (Bragg‐Brentano monochromated Cu Kα radiation λ = 1.54056 Å). The simulated PXRD patterns were generated from the corresponding MOF CIF files using Mercury software.

#### 4.2.2 Nitrogen Adsorption

Gas adsorption analyses were performed on a Quantachrome Quadrasorb‐SI automatic volumetric gas adsorption analyzer. A liquid nitrogen bath (77 K) and ultra‐high purity grade N_2_ and He (99.999%, Praxair) were used for measurements. The adsorption isotherms were collected after evacuating the samples at a temperature of 120°C for 12 h to ensure the desorption of any residual solvent molecules remaining after synthesis.

#### 4.2.3 ^1^H NMR Analysis of Digested Samples

^1^H NMR spectra were acquired on a Bruker Avance II 500 MHz spectrometer at 297 K. The samples were prepared by dissolving 5 mg of the sample in DMSO‐*d_6_* (560 µL), 40% hydrofluoric acid (20 µL), and D_2_O (20 µL).

#### 4.2.4 Electron Microscopy

TEM and STEM‐EDX measurements were performed on a multipurpose JEOL JEM‐2100 LaB_6_ equipment operated at an acceleration voltage of 200 kV. The samples were prepared for all measurements by dispersing the samples in ethanol using a sonic bath, followed by drop casting onto carbon film on a nickel grid.

#### 4.2.5 Thermogravimetric Analysis (TGA)

TGA measurements were carried out on a Mettler Toledo TGA/DSC 3+ HT/1600 apparatus with air zero as the sample gas. The sample was heated from 25 to 800°C with a ramp rate of 5°C/min.

#### 4.2.6 Inductively Coupled Plasma‐Atomic Emission Spectroscopy (ICP–OES)

The amounts of Cu in the samples were analyzed by an ICP–OES spectroscope (Optima 7000 DV, Perkin Elmer). The samples were digested as follows: 5 mg of the sample was dispersed in a solution mixture of nitric acid (2.25 mL) and hydrogen peroxide (0.25 mL), which was then heated to 185°C in an oven for 5 h. The solution mixture was adjusted to 25 mL with DI water in a 25 mL volumetric flask before measurement.

#### 4.2.7 In Situ Diffuse Reflectance FTIR Spectroscopy (DRIFTS)

The Thermo Scientific Nicolet iS50 spectrometer was used for the FTIR spectroscopy measurements. The spectrometer collects data in the mid‐IR, far‐IR, and near‐IR spectral ranges. Operando DRIFTS measurements were performed using a Praying Mantis High Temperature Reaction Chamber connected to the spectrometer. The setup allowed measurements at controlled temperatures and under a continuous flow of reaction gases. The spectrometer is equipped with a mercury–cadmium telluride (MCT) detector, which provides high sensitivity, fast response time, and good resolution. The DRIFTS measurements were recorded using Thermo Scientific OMNIC software, and data analysis was carried out with OMNIC Macros software according to Kubelka–Munk Unit (KMU) conversion and following similar procedures reported earlier. The value at a given frequency is determined by Equations 1 and 2. In these equations, R represents the normalized reflectance of the catalyst (I_sample_) during CO_2_ reduction, while I_background_ denotes the reflectance intensity of the sample under Ar, which corresponds to the background reflectance intensity. The reductively pre‐treated and reaction spectra are corrected according to the KMU procedure by using a background spectrum, recorded under a flow of Ar using KBr powder.

(1) $$\mathrm{KMU}=\frac{{\left(1-R\right)}^{2}}{2R}$$

(2) $$R=\frac{I_{\mathrm{sample}}}{I_{\mathrm{background}}}$$

#### 4.2.8 In Situ Diffuse Reflectance Ultraviolet–Visible Spectroscopy (DR‐UV–Vis)

A Shimadzu UV‐2600 spectrophotometer was used to record the DR‐UV–vis spectra. The spectrophotometer features a single monochromator and an 185–900 nm spectral range. Measurements of solid samples were carried out using the Praying Mantis chamber. After baseline correction, fresh or spent catalysts were introduced into the Praying Mantis chamber, and their DR‐UV–Vis spectra were collected and processed using LabSolutions UV–Vis software. The catalysts were characterized before and during the reductive treatment. First, the sample was purged with Ar (30 mL min^−1^), followed by a reduction step in (a gas mixture of 10% H_2_/Ar (30 mL min^−1^) for 2 h.

#### 4.2.9 Zeta Potential Measurements

Zeta potential measurements were performed using a Zetasizer Ultra Red equipment coupled with an MPT‐3 multi‐purpose titrator (Malvern Panalytical Ltd.) [46]. The instrument operates based on the principle of Electrophoretic Light Scattering (ELS), utilizing the M3‐PALS technique to ensure high precision in zeta potential measurements. For the measurement process, 50 mg of the catalyst was dispersed in 50 ml distilled water and was then sonicated for 15 min. Afterwards, 2 mL of the mixture was diluted with 10 mL distilled water. The whole amount was placed in the Zetasizer cell for measurement. The data were recorded using the ZS Xplorer Software.

#### 4.2.10 X‐Ray Absorption Spectroscopy (XAS)

Cu K‐edge XAS was measured at BL 5.2 (SUT‐NANOTEC‐SLRI), the SLRI Thailand storage ring. A Ge (220) double‐crystal monochromator with ΔE/E = 2 × 10^−4^ was used for scanning the synchrotron beam. Energy calibration was achieved using Cu metal foil at 8979 eV. For the in situ measurements, the powder catalyst was pelletized into a self‐standing disc with a diameter of 1.5 cm. The disc was positioned inside a custom‐made quartz tube. The quartz tube is equipped with gas inlet and outlet ports and Kapton windows for measurements in transmission mode. The quartz tube is heated using a tubular furnace. All spectra were acquired in fluorescence detection mode. The XANES region was scanned with a step size of 0.02 eV, and the EXAFS region was measured up to k = 14 Å^−1^ to ensure sufficient structural information. EXAFS data were processed with Athena and fitted with Artemis, both from the IFEFFIT package. All fits were conducted in k (2.5–11.5 Å^−1^) and R (1–3.8 Å) spaces. All EXAFS fitting parameters were optimized freely, using CuO and Cu_2_O CIF files as models.

### 4.3 Catalytic Experiments

#### 4.3.1 Gas Phase Catalysis

Catalytic tests at 20 and 50 bar were conducted in a setup with one fixed‐bed continuous‐flow reactor (276 mm in length with an external diameter of 7.6 mm). The amount of the catalyst was fixed to 300 mg. It was embedded between two layers of quartz wool and quartz particles. The feed gas flow (H_2_/CO_2_/N_2_ = 9/3/1) was adjusted to achieve a GHSV value of 7000 mln h^−1^ g_cat._^−1^. Prior to the reaction, the catalyst pretreatment program consisted of a drying step at 250°C under He flow of 30 mL min^−1^ followed by a reduction step using pure H_2_ at 250°C (30 mln/min). The feed gas mixture and the reaction products were analyzed with an on‐line gas chromatograph (Shimadzu GC‐2010 Plus) equipped with FID and TCD as well as Plot Q (for methanol, ethanol, 1‐ or 2‐propanol, C1−C3 hydrocarbons, acetone, dimethyl ether, and methyl formate) and MolSieve 5A columns (for H_2_, O_2_, N_2_, and CO). CO_2_ conversion (X(CO_2_)), CH_3_OH selectivity (S(CH_3_OH)), CO selectivity (S(CO)), CH_4_ selectivity (S(CH_4_)), C_2_H_5_OH selectivity (S(C_2_H_5_OH)), and C_2_H_6_ selectivity (S(C_2_H_6_)) were calculated according to Equations 3−8, respectively, where 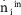 and 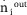 stand for the molar flows of gaseous components at the reactor inlet and outlet, respectively.

(3)

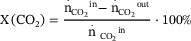



(4)

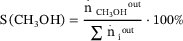



(5)

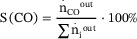



(6)

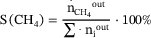



(7)

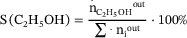



(8)

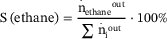




#### 4.3.2 Catalytic Measurements in Liquid Phase

Catalytic activity experiments in the liquid phase were carried out in batch mode using a Parr autoclave reactor, which is made of stainless steel and supports high pressure and temperature with a volume of 100 mL. The autoclave was equipped with a stir bar to mix the reactants. Glass tube inserts were used to serve as a barrier between the reactants and the stainless steel to prevent any contamination or undesired chemical interactions. The steps of the experiments are done according to the following sequence: at first, 100 mg of the reduced catalyst was dispersed in 10 mL of distilled water within the glass tube housed inside the reactor. Secondly, the autoclave loaded with the catalyst was purged with Ar gas 3 times to remove any residual air. Thirdly, the reactor was filled with carbon dioxide and hydrogen in a 1:3 ratio with a total pressure of 20 bar. Then, the temperature of the autoclave was increased to 175°C and the reactants were stirred at a slow speed, with a rotation rate of 100 rpm. Once the autoclave's temperature had reached 175°C, the stirring speed was increased to 500 rpm, and the timer was started to record the reaction time (18 h). Finally, after the reaction was done, the autoclave was cooled down to room temperature and the stirrer was turned off. The gas phase and liquid phase were then collected for quantitative analysis. The same process was used to make a blank measurement without a catalyst to serve as a reference for CO_2_ conversion. The reaction liquids were filtered and analyzed using a gas chromatography‐mass spectrometry (GC‐MS) system (GCMS‐GP2010 Ultra, Shimadzu) to identify and quantify products directly after the reaction. The reaction liquids separated after reaction were diluted using dimethyl sulfoxide (DMSO) and diethylene glycol dibutyl ether as internal standards for quantitative analysis. Calibration curves for methanol were prepared externally in the same solvent mixture over a concentration range from 0.01 to 1.0 M. All reaction samples were appropriately diluted in the same solvent prior to analysis to ensure accurate quantification. Gas‐phase products were analyzed by GC (GC‐7681, Agilent) for each experiment. Blank experiments of the catalyst in water and using Ar as filling gas were done to ensure there are no side products from catalyst decomposition during catalytic measurements. Based on these measurements, CO_2_ conversion (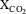) was calculated based on the pressure decrease observed after catalysis, using blank reactors without catalyst as references (Equation 9). P_0_ represents the CO_2_ pressure measured in a blank reactor without catalyst after heating and pressurization to the same conditions as the catalytic run, and P_x_ represents the CO_2_ pressure measured after completion of reaction.

The product selectivities in the gas and liquid phases were estimated according to equations ((10), (11), (12)). In these equations X_i_ stands for the yield fraction of the product i in the gas phase with carbon number n_i_. S_l_ stands for total product selectivity in liquid for different products having individual selectivities S_i_ (selectivity of all products in gas and liquid phases = 100%). x_i_ is the mol fraction of the product j in the liquid phase with carbon number = v_i_.

(9)

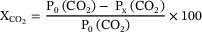



(10)

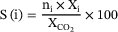

(11) $$S_{l}=100-\sum S_{i}\;$$

(12) $$S\left(j\right)=\frac{v_{j}\times x_{j}}{\sum v_{j}\times x_{j}}\times S_{l}\;$$

### 4.4 Statistical Analysis

Catalytic data: All measurements were performed in duplicate. Limited deviation was observed between measurements. No data transformation or normalization was applied. Outliers were not excluded. Data are presented as mean ± standard deviation in the text and Figures 6 and 7. Due to the limited sample size (*n* = 2), no inferential statistical tests were performed, and the results are reported descriptively. All statistical calculations were performed using Microsoft Excel and plotted using Origin. The statistical formula used for calculating the standard deviation is indicated in the Equation:

(13) $$s=\sqrt{\frac{1}{n-1}\sum_{i=1}^{n}{\left(x_{i}-\bar{x}\right)}^{2}}$$

*s* = sample standard deviation, *n* = number of measurements, *x_i_* = individual measurement $\bar{x}$ = mean value

For XAS, data were collected with a minimum of three scans to ensure sufficient signal‐to‐noise ratio and allow for a reasonable fit of the EXAFS data. The collected scans were merged prior to analysis using the Athena and Artemis software packages within the Demeter framework. Standard fitting procedures were applied in k‐space over the range of *k* = 2.5–10 Å^−1^. The fitting quality was evaluated using several statistical criteria, including an amplitude reduction factor (S_0_^2^) between 0.7 and 1.2, energy shift (ΔE_0_) values below 10 eV, and a disorder parameter(related to DWF) (σ^2^) less than 0.02 Å^2^. In addition, the number of fitting variables was kept lower than the number of independent parameters to avoid overfitting. The goodness‐of‐fit and R‐factor values were used to guide the fitting procedure and ensure statistically reliable results, with acceptable R‐factor values maintained below 0.03.

For DRIFTS measurements, spectra were collected using a KBr background reference. No post‐processing treatment was applied to the obtained spectra. The reductively pre‐treated and reaction spectra are corrected according to the KMU procedure by using a background spectrum, recorded on KBr powder under a flow of Ar (30 mL min^−1^).

For DR UV–Vis measurements, spectra were recorded from 800 to 200 nm with a step interval of 0.2 nm. Baseline correction was performed prior to sample measurements. Each spectrum acquisition required approximately 14 min. No normalization or additional post‐processing treatment was applied, and the spectra are presented as directly acquired from the instrument.

## Conflicts of Interest

The authors declare no conflicts of interest.

## Supporting information




**Supporting File**: smtd70882‐sup‐0001‐SuppMat.docx.

## Data Availability

The data that support the findings of this study are available in part in the Supporting Information, and any additional data will be made available upon request from the corresponding authors.
