# Supplementary material for: Regulating Copper Nuclearity in Metal–Organic Frameworks for the Hydrogenation of Carbon Dioxide
Source: Small Methods. 2026 Aug 17;10(18):e70882. doi: 10.1002/smtd.70882 (PMC13599685; doi:10.1002/smtd.70882)
Supplement: Supplementary file 1 [file SMTD-10-e70882-s001.docx]

**Supporting Information**

**Regulating Copper Nuclearity in Metal–Organic Frameworks for the Hydrogenation of Carbon Dioxide**

Soufiane Bahou,^1^ Chanokporn Kosri,^2^ Ashour A. Ahmed^,1,3^ Hesham Mena,^1^ Riya Sehrawat,^1^ Sebastian Wohlrab,^1^ Thanapa Numpilai,^4^ Pongtanawat Khemthong,^2^ Thongthai Witoon,^5^ Kajornsak Faungnawakij,^2^ Bunyarat Rungtaweevoranit^2^*, Ali M. Abdel-Mageed^1,6^*

^1^Leibniz Institute for Catalysis (LIKAT), D-18059 Rostock, Germany

^2^National Nanotechnology Center (NANOTEC), National Science and Technology Development Agency (NSTDA), Pathum Thani 12120, Thailand

^3^ CASUS - Center for Advanced Systems Understanding, Helmholtz-Zentrum Dresden-Rossendorf e.V. (HZDR), Untermarkt 20, Görlitz D-02826, Germany

^4^ Department of Environmental Science, Faculty of Science and Technology, Thammasat University, Pathumthani, 12120, Thailand

^5^ Center of Excellence on Advanced Adsorbents and Catalysts for Carbon Capture and Utilization, Department of Chemical Engineering, Faculty of Engineering, Kasetsart University, Bangkok 10900, Thailand

^6^ Department of Chemistry, Faculty of Science, Cairo University, Giza 12613, Egypt.

* Corresponding authors: ali.abdelmageed@catalysis.de and bunyarat.run@nanotec.or.th

**List of Supporting Information Figures**

| **Item** | **Description** | **Page** |
| --- | --- | --- |
| Figure S1 | PXRD (reduced Cu–MOF catalysts) | S3 |
| Figure S2 | N_2_ isotherms (Cu/UiO-66 vs UiO-66) | S4 |
| Figure S3 | N_2_ isotherms (Cu/MOF-808 vs MOF-808) | S5 |
| Figure S4 | N_2_ isotherms (reduced Cu–MOFs) | S6 |
| Figure S5 | ¹H NMR spectra (UiO-66) | S7 |
| Figure S6 | ^1^H NMR spectra (Cu_x_/UiO-66) | S8 |
| Figure S7 | ^1^H NMR spectra (Cu_1_/UiO-66) | S9 |
| Figure S8 | ^1^H NMR spectra (MOF-808) | S10 |
| Figure S9 | ^1^H NMR spectra (Cu_x_/MOF-808) | S11 |
| Figure S10 | ^1^H NMR spectra (Cu_1_/MOF-808) | S12 |
| Figure S11 | TGA profile (UiO-66 series) | S13 |
| Figure S12 | TGA profile (MOF-808 series) | S14 |
| Figure S13 | TEM results (fresh catalysts) | S15 |
| Figure S14 | TEM results (reduced catalysts) | S16 |
| Figure S15 | TEM results (spent catalysts) | S17 |
| Figure S16 | Optimized structures of Cu_x_/UiO-66 and Cu_x_/MOF808 | S18 |
| Figure S17 | Optimized structures of Cu_1_/UiO-66 and Cu_1_/MOF808 | S19 |
| Figure S18 | Zeta potential as a function of pH | S20 |
| Figure S19 | PXRD pattern (fresh Cu/ZnO-Al_2_O_3_) | S21 |
| Figure S20 | PXRD pattern (reduced Cu/ZnO-Al_2_O_3_) | S22 |
| Figure S21 | CO_2_ hydrogenation at 200 °C on Cu1/UiO-66 catalysts | S23 |
| Figure S22 | CO_2_ hydrogenation at 225 °C on Cu1/UiO-66 catalysts | S24 |
| Figure S23 | CO_2_ hydrogenation at different temperatures on Cu_x_/UiO-66 catalyst | S25 |
| Figure S24 | CO_2_ hydrogenation at different temperatures on Cu_x_/MOF808catalyst | S26 |
| Figure S25 | GC-MS data (Cu_1_/UiO-66) | S27 |
| Figure S26 | GC-MS data (Cu_1_/MOF-808) | S28 |
| Figure S27 | PXRD data (Cu_1_/UiO-66, spent) | S29 |
| Figure S28 | PXRD data (Cu_1_/MOF-808, spent) | S30 |
| Figure S29 | PXRD data (Cu_x_/UiO-66, spent) | S31 |
| Figure S30 | PXRDdata (Cu_x_/MOF-808, spent) | S32 |
| Figure S31 | STEM-EDS (Cu_1_/UiO-66) | S33 |
| Figure S32 | STEM-EDS (Cu_1_/MOF-808) | S34 |
| Figure S33 | STEM-EDS (Cu_x_/UiO-66) | S35 |
| Figure S34 | STEM-EDS (Cu_x_/MOF-808) | S36 |
| Figure S35 | DRIFTS (CO_2_ reduction intermediates) | S37 |
| References |  | S38 |

**Figure S1.** PXRD patterns of the reduced Cu-MOF catalysts: Cu_x_/UiO-66, Cu_1_/UiO-66, Cu_x_/MOF-808 and Cu_1_/MOF-808.

**Figure S2.** N_2_ adsorption-desorption isotherms of Cu immobilized on UiO-66 compared to bare UiO-66 at 77 K with adsorption and desorption points represented by closed circles and open circles, respectively.

**Figure S3.** N_2_ adsorption-desorption isotherms of Cu immobilized on MOF-808 compared to bare MOF-808 at 77 K with adsorption and desorption points represented by closed circles and open circles, respectively.

**Figure S4.** N_2_ adsorption-desorption isotherms of the reduced samples; Cu_x_/UiO-66, Cu_1_/UiO-66, Cu_x_/MOF-808 and Cu_1_/MOF-808; at 77 K with adsorption and desorption points represented by closed circles and open circles, respectively.

**Figure S5:** ^1^H NMR spectra of the as prepared digested UiO-66 metal-organic framework.

**Figure S6.** ^1^H NMR spectrum of the as-prepared digested Cu_x_/UiO-66 metal-organic framework.

**Figure S7.** ^1^H NMR spectrum of the as-prepared digested Cu_1_/UiO-66 metal-organic framework.

**Figure S8.** ^1^H NMR spectrum of the as-prepared digested MOF-808 metal-organic framework.

**Figure S9.** ^1^H NMR spectrum of as-prepared digested Cu_x_/MOF-808 metal-organic framework.

**Figure S10.** ^1^H NMR spectrum of the as-prepared digested Cu_1_/MOF-808 metal-organic framework

**Figure S11.** TGA profiles of UiO-66, Cu_x_/UiO-66 and Cu_1_/UiO-66 measured in air.

**Figure S12.** TGA profiles of MOF-808, Cu_x_/MOF-808 and Cu_1_/MOF-808 of measured in air.

**Figure S13.** TEM images of as-synthesized catalysts: Cu_x_/UiO-66 (A), Cu_1_/UiO-66 (B), Cu_x_/MOF-808 (C), and Cu_1_/MOF-808 (D).

**Figure S14.** TEM images of reduced catalysts: Cu_x_/UiO-66 (A), Cu_1_/UiO-66 (B), Cu_x_/MOF-808 (C), and Cu_1_/MOF-808 (D).

**Figure S15.** TEM images of catalysts after reaction: Cu_x_/UiO-66 (A), Cu_1_/UiO-66 (B), Cu_x_/MOF-808 (C), and Cu_1_/MOF-808 (D).

**Figure S16**: DFT optimized structure of Cu3 clusters anchored into the UiO-66 (a) and the MOF808(b) frameworks ( Color code: Cu: blue; Zr: green; C: black; O: red; H:white). For details on theoretical calculations see References. [1, 2]

**Figure S17**: DFT optimized structure of isolated Cu_1_ sites anchored into the UiO-66 (a) and two structures of Cu_1_ anchored into MOF808 (b, c) framework ( Color code: Cu: blue; Zr: green; C: black; O: red; H:white). For details on theoretical calculations see References. [1, 2]

**Figure S18.** pH (2-10.5) dependence of zeta potential for the Cu-free MOFs and the Cu metalated MOFs (a: UiO-66; b: Cu_x_/UiO-66; c: MOF-808; d: Cu_x_/MOF-808).

**Figure S19.** PXRD patterns of the fresh commercial Cu/ZnO-Al_2_O_3_ catalyst.

**Figure S20.** PXRD patterns of reduced commercial Cu/ZnO-Al_2_O_3_ catalyst.

**Figure S21.** The CO_2_-conversions and selectivities for CH_3_OH, CO and CH_4_ in CO_2_ hydrogenation over Cu_1_/UiO-66 catalyst tested at 200 °C and 50 bar using a feed with the ratio of H_2_/CO_2_/N_2_ = 9:3:1.

**Figure S22.** The CO_2_-conversions and selectivities for CH_3_OH, CO and CH_4_ in CO_2_ hydrogenation over Cu_1_/UiO-66 catalyst tested at 225 °C and 50 bar using a using a feed with the ratio of H_2_/CO_2_/N_2_ = 9:3:1.

**Figure S23**: Time-on-stream reaction rate on Cu_x_/UiO-66 catalyst at 3 different temperatures and total pressure of 20 bar of CO2/H2 (1:3) and using 100 mg of the catalyst.

**Figure S24**: Time-on-stream reaction rate on Cu_x_/MOF808 catalyst at 3 different temperatures and total pressure of 20 bar of CO_2_/H_2_ (1:3) and using 100 mg of the catalyst.

**Figure S25**. GC-MS chromatogram collected for the CO_2_ hydrogenation at 175 °C / 20 bar in water on the Cu_1_/UiO-66 catalysts.

**Figure S26**: GC-MS chromatogram collected for the CO_2_ hydrogenation at 175 °C / 20 bar in water on the Cu_1_/MOF-808 catalysts.

**Figure S27.** PXRD patterns of the Cu_1_/UiO-66 after CO_2_ hydrogenation reaction (CO_2_/N_2_ = 9/3/1) in different temperatures at 20 bar compared to the as-prepared catalyst.

**Figure S28.** PXRD patterns of the Cu_1_/MOF-808 CO_2_ hydrogenation reaction (H_2_/CO_2_/N_2_ = 9/3/1) at different temperatures at 20 bar, compared to the as-prepared catalyst.

**Figure S29.** PXRD patterns of the Cu_x_/UiO-66 after CO_2_ hydrogenation (H_2_/CO_2_/N_2_ = 9/3/1) at different temperatures at 20 bar, compared to the as-prepared catalyst.

**Figure S30.** PXRD patterns of the Cu_x_/MOF-808 after CO_2_ hydrogenation (H_2_/CO_2_/N_2_ = 9/3/1) at different temperatures at 20 bar, compared to the as-prepared catalyst.

**Figure S31:** STEM-EDS analysis of Cu_1_/UiO-66 catalysts after different stages (a: fresh; b: reduced at 225 °C; c: spent in CO_2_-reaction at 225 °C / 20 bar).

**Figure S32.** STEM-EDS analysis of Cu_1_/MOF-808 catalysts after different stages (a: fresh; b: reduced at 225 °C; c: spent in CO_2_-reaction at 225 °C / 20 bar).

**Figure S33.** STEM-EDS analysis of Cu_x_/UiO-66 catalysts after different stages (a: fresh; b: reduced at 225 °C; c: spent in CO_2_-reaction at 225 °C / 20 bar).

**Figure S34.** STEM-EDS analysis of Cu_x_/MOF-808 catalysts after different stages (a: fresh; b: reduced at 225 °C; c: spent in CO_2_-reaction at 225 °C / 20 bar).

**Figure S35.** Kubelka-Munk converted DRIFT spectra of different catalysts (a: Cu_1_/UiO-66; b: Cu_x_/UiO-66; c: Cu_x_/MOF-808) in the spectral range from 2215 and 1950 cm^-1^ after pretreatment (black spectrum) and during CO_2_ reduction (CO_2_/H_2_ = 1:3 at 20 bar) at different temperatures, carbonyl C-O region.

**References**

[1] S. Bahou, R. Sehrawat, B. Rungtaweevoranit, A.M. Abdel-Mageed, A.A. Ahmed, Fingerprinting Single and Clustered Cu Sites in Metalated MOF Catalysts via TD-DFT and In Situ Diffuse Reflectance UV–Vis Spectroscopy, ACS Appl. Mater. Interf., 17 (2025) 52840–52853.

[2] D. Makhmutov, B. Rungtaweevoranit, A.A. Ahmed, K. Faungnawakij, M. Al-Yusufi, E. Salaya, S. Wohlrab, U. Armbruster, A.M. Abdel-Mageed, Catalytic and kinetic isotope effect studies of CO2 reduction on Cu-Metalated UiO-66 Metal-Organic framework, Appl. Sur. Sci., 688 (2025) 162323.
